# Dynamics of the Eukaryotic Replicative Helicase at Lagging-Strand Protein Barriers Support the Steric Exclusion Model

**DOI:** 10.1016/j.celrep.2019.01.086

**Published:** 2019-02-19

**Authors:** Hazal B. Kose, Nicolai B. Larsen, Julien P. Duxin, Hasan Yardimci

**Affiliations:** 1 Single Molecule Imaging of Genome Duplication and Maintenance Laboratory, The Francis Crick Institute, NW1 1AT London, UK; 2 Novo Nordisk Foundation Center for Protein Research, Faculty of Health and Medical Sciences, University of Copenhagen, DK-2200 Copenhagen, Denmark

**Keywords:** eukaryotic DNA replication, CMG, replicative helicase, DNA-protein crosslink, single-molecule imaging, steric exclusion

## Abstract

Progression of DNA replication depends on the ability of the replisome complex to overcome nucleoprotein barriers. During eukaryotic replication, the CMG helicase translocates along the leading-strand template and unwinds the DNA double helix. While proteins bound to the leading-strand template efficiently block the helicase, the impact of lagging-strand protein obstacles on helicase translocation and replisome progression remains controversial. Here, we show that CMG and replisome progressions are impaired when proteins crosslinked to the lagging-strand template enhance the stability of duplex DNA. In contrast, proteins that exclusively interact with the lagging-strand template influence neither the translocation of isolated CMG nor replisome progression in *Xenopus* egg extracts. Our data imply that CMG completely excludes the lagging-strand template from the helicase central channel while unwinding DNA at the replication fork, which clarifies how two CMG helicases could freely cross one another during replication initiation and termination.

## Highlights

• The CMG helicase can bypass a lagging-strand DNA-protein crosslink without stalling
• A methyltransferase on the lagging strand stalls CMG by stabilizing duplex DNA
• Single-molecule detection of CMG pausing at a lagging-strand methyltransferase
• Replisome progression matches the dynamics of purified CMG at identical roadblocks

## Graphical Abstract

**Figure:**
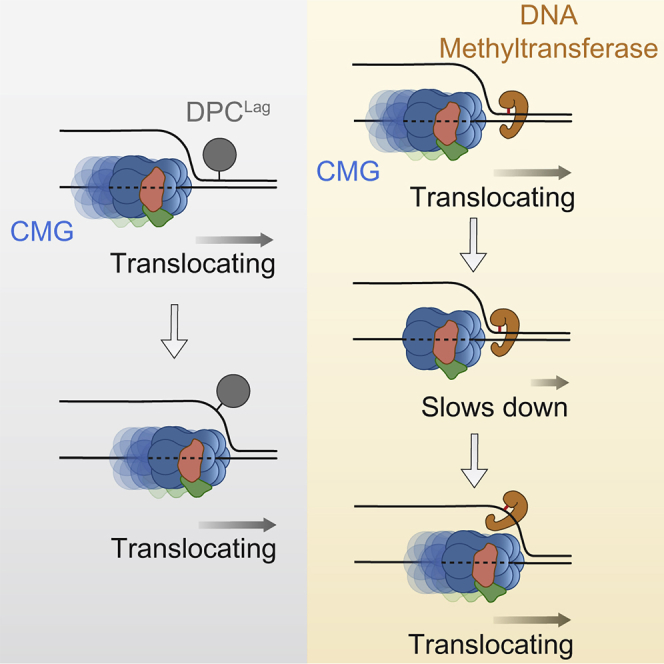

## Teaser

During DNA replication, the CMG complex unwinds the double helix and must overcome protein barriers on DNA. Kose et al. show that proteins that interact exclusively with the lagging-strand template are bypassed by the helicase without stalling, implying that the lagging-strand template is completely excluded from CMG during unwinding.

## Introduction

In eukaryotic cells, many origin sites on DNA are “licensed” (Blow and Laskey, 1988, Chong et al., 1995, Kubota et al., 1995, Madine et al., 1995) for replication in the Gap 1 (G1) phase by the loading of hetero-hexameric Minichromosome maintenance (Mcm) 2-7 complexes through the collective actions of Origin Recognition Complex (ORC), Cdt1, and Cdc6 (Coster and Diffley, 2017, Coster et al., 2014, Evrin et al., 2009, Kang et al., 2014, Remus et al., 2009, Ticau et al., 2015). Mcm2-7 complexes, which assemble around double-stranded DNA (dsDNA) as double hexamers, are called pre-replication complexes (preRCs). PreRCs remain inactive until cells enter into the Synthesis (S) phase, after which two protein kinases, namely, Cyclin-Dependent Kinase (CDK) and Dbf4-Dependent Kinase (DDK), are involved in activating the helicase. While CDK phosphorylates Sld2 and Sld3, DDK directly phosphorylates Mcm2-7 for the recruitment of Cdc45 and the GINS complex (Douglas et al., 2018, Heller et al., 2011, Labib, 2010, Muramatsu et al., 2010, Sheu and Stillman, 2006, Tanaka et al., 2007, Zegerman and Diffley, 2007). Subsequent to its association with Cdc45 and GINS, the two Mcm2-7 complexes, whose N-termini initially face each other, are activated, and the CMG (Cdc45/Mcm2-7/GINS) complexes unwind the DNA (Costa et al., 2011, Douglas et al., 2018, Ilves et al., 2010, Moyer et al., 2006, Yardimci et al., 2010). Recruitment of a number of other factors establishes the replisome complex, and the unwound DNA is replicated through the synthesis of the leading and lagging strands by polymerase epsilon and delta, respectively (Lujan et al., 2016).

In S phase, replisome progression is challenged by various DNA lesions and DNA-protein complexes, including nucleosomes, transcription machinery, and DNA-protein crosslinks (DPCs). As the replicative helicase forms the core of the replisome, its engagement with DNA and DNA-protein complexes has been the subject of intense study. Most replicative helicases unwind DNA via the steric exclusion mechanism where the helicase translocates along one strand while excluding the other entirely from the helicase central channel. As a consequence, although replicative helicases either arrest at or remove protein obstacles bound to the translocation strand, they are able traverse bulky barriers that interact with the displaced strand, as shown for T7 gp4, DnaB, E1, and Simian Virus 40 (SV40) large T-antigen (LTag) (Egelman et al., 1995, Enemark and Joshua-Tor, 2006, Jeong et al., 2004, Kaplan, 2000, Kaplan et al., 2003, Lee et al., 2014, Yardimci et al., 2012a).

How the eukaryotic CMG helicase interacts with protein barriers during unwinding is less well understood. The Mcm4/6/7 complex arrests at a leading-strand DPC and is able to bypass a lagging-strand DPC (Nakano et al., 2013), consistent with the steric exclusion model. In line with this observation, replication fork progression in *Xenopus laevis* egg extracts is blocked by protein barriers attached to the leading- but not to the lagging-strand template, suggesting that the CMG ring encircles only the leading-strand template during unwinding (Fu et al., 2011). However, the presence of many other protein factors in egg extracts makes it possible that bypass of lagging-strand barriers by CMG is facilitated by accessory proteins. In fact, given sufficient time, CMG is able to bypass even a leading-strand protein roadblock in egg extracts with the help of additional factors (Sparks et al., 2019). In support of the possibility that bypass of lagging-strand obstacles by CMG is promoted by other replisome components, inhibition of DNA unwinding by recombinant-purified *Saccharomyces cerevisiae* CMG (*Sc*CMG) complex was reported in the presence of lagging-strand protein blocks (Langston and O’Donnell, 2017). Furthermore, the structure of *Sc*CMG on a preformed fork DNA (henceforth referred to as fork DNA) substrate containing roadblocks displayed a short segment of dsDNA entering into the MCM pore (Georgescu et al., 2017). Together, these results led to the proposal of a modified steric exclusion model where the lagging-strand template initially enters the central ring and subsequently makes either a U-turn and is extruded out the same channel (Langston and O’Donnell, 2017) or exits through the protrusions within the MCM zinc finger domains (O’Donnell and Li, 2018). In addition, Mcm10 was reported to relieve obstruction of *Sc*CMG helicase activity by lagging-strand blocks, suggesting that it may open the CMG ring upon roadblock collision (Langston et al., 2017). In egg extracts, a dual biotin-streptavidin complex (Fu et al., 2011) as well as a covalently trapped methyltransferase (Duxin et al., 2014) on the lagging-strand template led to transient stalling of nascent leading strands at a distance matching the CMG footprint (Fu et al., 2011), raising the possibility that CMG goes through a conformational change, such as opening its ring, to bypass the protein barrier. However, whether Mcm10 is needed for traversal of lagging-strand barriers in egg extracts has not yet been tested.

It is essential to understand the dynamics of CMG at DNA-protein complexes, which is one of the main determinants of how replication forks navigate through the protein-rich chromatin environment. Using ensemble and single-molecule biochemistry, we investigated the outcome of CMG collision with strand-specific protein roadblocks. We show that proteins that exclusively interact with the lagging-strand template do not impact CMG or replisome progression, supporting strand exclusion as the preferred mechanism of DNA unwinding by CMG.

## Results

### Biotin–Streptavidin Complex on the Leading-Strand Template Inhibits DNA Unwinding by CMG

To understand how CMG interacts with DNA at the replication fork, we purified *Drosophila melanogaster* CMG (*Dm*CMG) by overexpressing all 11 subunits of the complex in insect cells (Figure S1A) (Ilves et al., 2010). The recombinant CMG unwinds fork DNA substrates in the 3′-to-5′ direction (Georgescu et al., 2014, Ilves et al., 2010, Kang et al., 2012, Moyer et al., 2006, Petojevic et al., 2015). Although a fork DNA substrate bearing 40-nucleotide (nt) poly-T sequence (dT_40_) on both single-stranded DNA (ssDNA) tails can bind two CMG complexes, replacing the 5′ lagging-strand arm with repeats of a GGCA sequence leads to only one CMG binding, as this sequence forms secondary hairpin-like structures preventing CMG assembly on the 5′ tail (Petojevic et al., 2015). Thus, to measure the helicase activity of CMG, we designed fork DNA substrates bearing a 3′ dT_40_ tail and 5′ d(GGCA) repeats, as well as a 60-base pair (bp) duplex region to be unwound.

To determine whether strand separation occurs inside or outside the helicase central channel, we examined the DNA unwinding activity of CMG on substrates, including a biotin-streptavidin roadblock attached to either the leading- or lagging-strand templates at the center of the duplex region. Although some replicative helicases are able to dislodge streptavidin (SA) from the translocation strand, such as E1 and LTag (Lee et al., 2014, Yardimci et al., 2012a), others are not strong enough to break biotin-streptavidin (bio-SA) interaction, as seen for DnaB (Kaplan, 2000). As CMG tracks along the leading-strand template, SA on this strand (SA^Lead^) should either stall the helicase or get displaced during unwinding. If the ss-dsDNA junction is buried inside the helicase ring, the SA attached to the lagging-strand template (SA^Lag^) is also expected to either block helicase activity or be dislodged by CMG. In contrast, if the lagging-strand template is completely excluded outside the CMG ring, SA^Lag^ should not influence CMG translocation, nor should CMG remove SA^Lag^.

We observed significant disengagement of SA attached to biotin on an internal thymidine base through a 6-carbon spacer (biotin-dT) on dsDNA (Figure S1B, lanes 1–6) but not ssDNA (Figure S1C, lanes 1–6), as reported previously (Brüning et al., 2016). We inserted a tetraethylene glycol (PEG4) chain between biotin and the thymidine base, which greatly reduced the release of SA from dsDNA when challenged with free biotin (Figure S1B, lanes 7–12). Given their stable SA binding, we used fork DNA substrates containing an internal biotin with an additional PEG4 spacer either on the leading- or lagging-strand templates to investigate the interaction of CMG with site-specific protein barriers.

**Figure 1:**
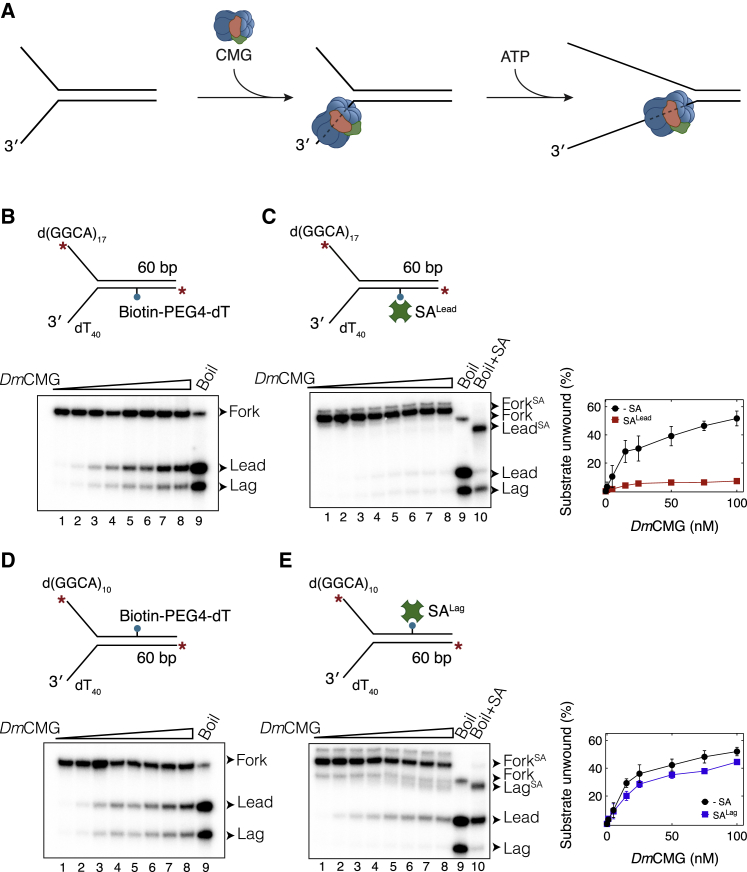
Interaction of *Dm*CMG with Strand-Specific Biotin-Streptavidin Complexes (A) Experimental approach used in unwinding assays. (B and C) *Dm*CMG-mediated unwinding of fork DNA templates in the (B) absence or (C) presence of SA^Lead^. Right panel shows percentage of substrate unwound as a function of *Dm*CMG concentration. The extended length on the 5′ tail (17 repeats of d(GGCA)) was necessary to discriminate SA-bound ssDNA (Lead^SA^) from naked dsDNA (Fork) when separated on polyacrylamide gel. (D and E) Unwinding of fork DNA templates in the (D) absence or (E) presence of SA^Lag^. Right panel shows percentage of substrate unwound as a function of *Dm*CMG concentration. In all gel images, lanes 1–8 correspond to reactions containing 0, 1, 5, 15, 25, 50, 75, and 100 nM *Dm*CMG. Lane 9 contains heat-denatured fork DNA that marks positions of the leading- (Lead) and lagging-strand (Lag) templates. Addition of SA to denatured DNA (lane 10) reveals positions of SA-bound leading- (Lead^SA^) and lagging-strand (Lag^SA^) templates. Fork substrates were labeled at both 5′ ends with ^32^P. The radiolabel is shown as a red asterisk. Data on the right panels correspond to mean ± SD from three independent experiments. See also Figure S1.

We examined the consequence of *Dm*CMG encountering a bio-SA complex on the leading-strand template. For efficient assembly of the helicase on the 3′ ssDNA overhang of the fork, we first incubated *Dm*CMG with DNA in the presence of ATPγS for 120 min (Petojevic et al., 2015). DNA unwinding was then initiated by adding ATP into the reaction (Figure 1A). After a 10-min incubation with ATP, the reaction was stopped, and DNA was separated on a non-denaturing gel. In the absence of SA, *Dm*CMG unwound 52% ± 5% of DNA, at 100 nM *Dm*CMG (Figure 1B), indicating that purified *Dm*CMG functions as an active helicase (Ilves et al., 2010). Importantly, the unwinding efficiency decreased to 7% ± 0.1% in the presence of SA^Lead^, suggesting that *Dm*CMG is unable to disrupt the bio-SA interaction (Figure 1C). The extent of unwinding did not increase in the presence of excess biotin, which would capture any SA removed from DNA (Figures S1D and S1E). This result supports that *Dm*CMG is not proficient at breaking the bio-SA linkage.

### CMG Bypasses a Lagging-Strand Biotin-Streptavidin Complex without Displacing Streptavidin

To determine whether DNA unwinding occurs externally or within the central channel of the helicase, we measured CMG-mediated unwinding of DNA templates containing SA^Lag^. If part of CMG encircles dsDNA at the fork, SA^Lag^ is expected to block helicase movement and, thus, DNA unwinding because CMG is not able to disrupt the bio-SA interaction (Figure 1C). DNA containing SA^Lag^ was unwound as efficiently as DNA lacking SA (Figures 1D and 1E), suggesting the helicase encircles only the leading-strand template in its central channel. To rule out the possibility that SA^Lag^ is dislodged by the helicase during unwinding, we added excess biotin to the reaction to sequester any released SA (Figure S1F). The fraction of SA-bound ssDNA remained the same in the presence of excess biotin (Figure S1F, right panel), indicating that CMG did not displace SA^Lag^. Therefore, our results strongly suggest that *Dm*CMG unwinds DNA via the steric exclusion mechanism.

**Figure 2:**
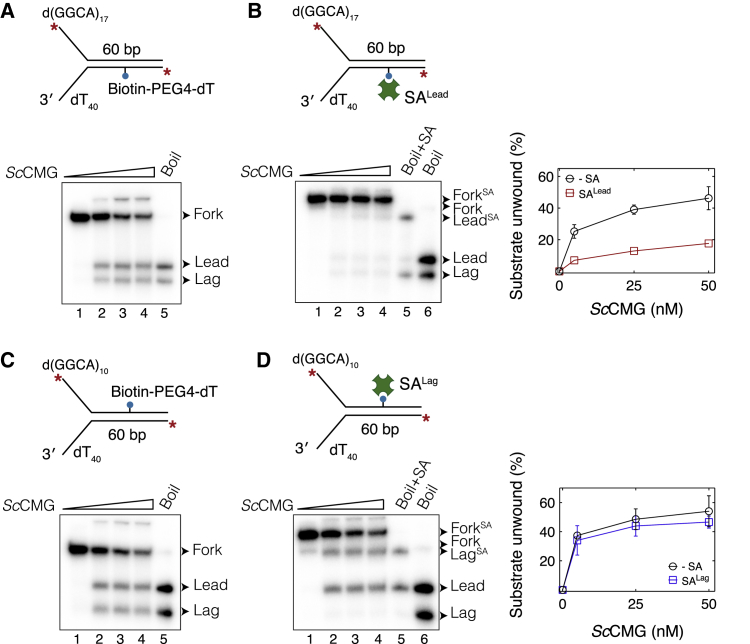
*Sc*CMG Bypasses a Biotin-Streptavidin Complex on the Excluded Strand (A and B) Unwinding of fork DNA by *Sc*CMG in the (A) absence or (B) presence of SA^Lead^. Right panel shows percentage of substrate unwound against *Sc*CMG concentration. (C and D) Unwinding of fork DNA by *Sc*CMG in the (C) absence or (D) presence of SA^Lag^. Right panel shows percentage of substrate unwound against *Sc*CMG concentration. In all gel images, lanes 1–4 correspond to reactions with 0, 5, 25, and 50 nM *Sc*CMG. In panels (B) and (D), heat-denatured fork DNA was incubated with SA (lane 5) revealing the positions of SA-bound leading- (Lead^SA^) and lagging-strand (Lag^SA^) templates. All fork DNA templates used in these assays were labeled at both 5′ ends with ^32^P. The radiolabel is shown as a red asterisk. Data represented here are mean ± SD from three independent experiments. See also Figure S2.

Previous biochemical assays performed with isolated *Sc*CMG showed that a single bio-SA block on the lagging-strand template caused 50% inhibition of helicase activity, whereas a double bio-SA complex on this strand inhibited DNA unwinding almost completely (Langston and O’Donnell, 2017). To determine whether the DNA translocation mechanism of *Sc*CMG differs from that of *Dm*CMG, we repeated unwinding assays by using purified recombinant *Sc*CMG (Zhou et al., 2017) and the same set of fork DNA templates bearing site-specific bio-SA blocks. As predicted, SA^Lead^ considerably hindered *Sc*CMG-mediated DNA unwinding (Figures 2A and 2B). When bound to the lagging-strand template, SA did not block the helicase activity of *Sc*CMG (Figures 2C and 2D). Furthermore, *Sc*CMG did not displace SA^Lag^ as the majority of unwound DNA retained SA on this strand in the presence of free biotin (Figures S2A and S2B). Our results suggest that, similar to *Dm*CMG, *Sc*CMG can efficiently bypass a lagging-strand protein barrier.

### A Protein Attached to Fork DNA Can Hinder CMG Binding

**Figure 3:**
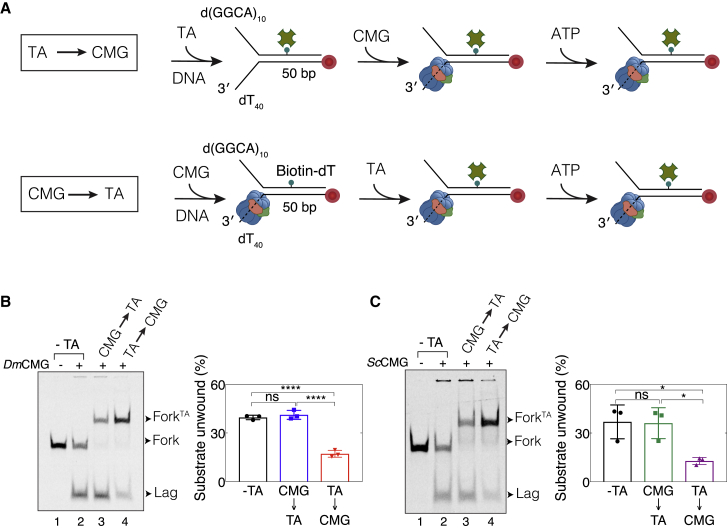
A Protein Attached to Fork DNA Can Hinder CMG Binding (A) Schematic representation of experimental approach used in unwinding assays. “CMG→TA” refers to the strategy where CMG was allowed to bind the fork substrate before addition of traptavidin (TA). “TA→CMG” corresponds to CMG binding to the fork DNA that was pre-bound to TA. (B and C) Unwinding of fork DNA bearing TA^Lag^ by (B) *Dm*CMG or (C) *Sc*CMG. Right panels show percentage of substrate unwound in each reaction. In all gel images, lane 1 corresponds to reaction lacking CMG, lane 2 to reaction lacking TA, lanes 3 and 4 to reactions including CMG and TA in different orders as indicated. All reactions included 5 nM DNA substrate and 50 nM helicase. DNA templates are Cy5 labeled at the 5′ end of the leading-strand template. Data represented here are mean ± SD from three independent experiments. n.s., not significant; ^∗^p < 0.05, ^∗∗^p < 0.01, ^∗∗∗^p < 0.001. See also Figure S3.

To determine the origin of helicase inhibition by a lagging-strand protein barrier in previous work (Langston and O’Donnell, 2017), we modified the fork template to match the sequence used by Langston and O’Donnell (2017). These templates contained a 50-bp duplex and a biotin on an internal thymidine base lacking a PEG4 linker on the lagging-strand template (Figure 3A). Because we observed significant dissociation of SA attached to an internal biotin-dT on dsDNA (Figure S1B), we used traptavidin (TA), a two-amino acid SA mutant with 10-fold lower biotin off-rate than SA (Chivers et al., 2010). TA showed minimal dissociation from dsDNA modified with biotin-dT (Figure S1G). TA attached to the lagging-strand template (TA^Lag^) led to a ∼50% drop in unwinding by *Dm*CMG and *Sc*CMG on this DNA substrate (Figures 3B and 3C, TA→CMG), closely matching previous results (Langston and O’Donnell, 2017, Langston et al., 2017). Given that biotin was attached to DNA by a PEG4 linker in our original templates (Figures 1 and 2), we considered the possibility that when bound to DNA by a short linker, TA may obscure efficient binding of CMG to fork DNA, causing inefficient unwinding. To test this idea, we changed the order of TA and CMG binding to DNA (Figure 3A). Strikingly, no inhibition was observed when either *Dm*CMG or *Sc*CMG was bound to the fork template before TA (Figures 3B and 3C, CMG→TA). To verify that TA bound to DNA before ATP addition, we added excess biotin into the reaction together with ATP. The level of TA-bound fork DNA (Fork^TA^) remained essentially the same in the presence of excess biotin (Figure S3), indicating that TA efficiently bound to DNA after CMG assembly and before unwinding was initiated. Together, our data strongly indicate that TA bound to internal biotin-dT without an additional PEG4 linker on the parental duplex of a fork DNA impairs the productive binding of CMG onto the fork, likely due to steric hindrance, and explains helicase inhibition by lagging-strand protein barriers observed previously (Langston and O’Donnell, 2017).

### Action of CMG at Covalent DPCs

Efficient traversal of lagging-strand protein barriers by CMG implies that the helicase ring does not encircle the lagging-strand template during translocation. However, one possibility is that part of the lagging-strand template at the fork resides within the CMG ring and that this strand is excluded only after the helicase encounters a protein roadblock on the same strand. If the replisome stalling in the presence of lagging-strand barriers observed in egg extracts (Duxin et al., 2014, Fu et al., 2011) is due to CMG encircling the non-translocation strand, isolated CMG would be expected to slow down at a lagging-strand obstacle before fully unwinding DNA in our assays. To test this idea, we investigated whether a lagging-strand protein barrier changes the kinetics of CMG-mediated DNA unwinding.

To analyze how a barrier on the non-translocation strand affects DNA unwinding in a time-course assay, we used a non-removable protein roadblock because SA gradually dissociates from biotin on dsDNA (Figure S1B). Therefore, we created site-specific covalent DNA-protein crosslinks (DPCs). To this end, SA was first functionalized with Dibenzocyclooctyl (DBCO) and covalently conjugated to azide-modified DNA via DBCO-azide-mediated copper-free click chemistry (Baskin et al., 2007, Jewett et al., 2010). To compare the stability of SA binding, DNA templates were subjected to 50°C for 10 min. Although SA dissociated from biotin on dsDNA even in the presence of a PEG4 linker at elevated temperatures, no release was observed on the substrate in which SA was crosslinked by click chemistry (clk-SA) (Figure S4A), as expected from a covalent bond.

**Figure 4:**
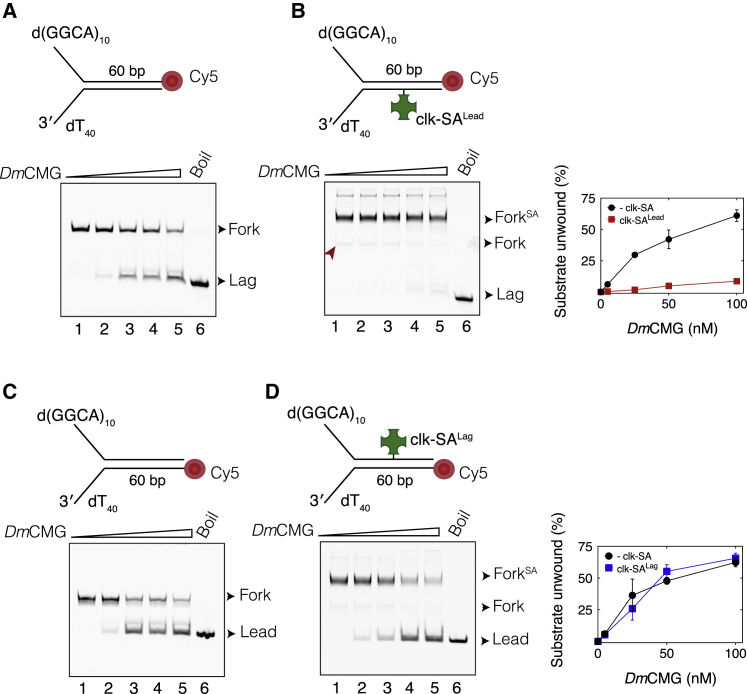
CMG Can Bypass a Covalent Lagging-Strand Protein Crosslink (A and B) *Dm*CMG-mediated unwinding of fork DNA in the (A) absence or (B) presence of clk-SA^Lead^. DNA contained a Cy5 at the 3′ end of the lagging-strand template. Weak unwinding that was observed on the clk-SA^Lead^-modified substrate could be attributed to trace amounts of non-conjugated DNA substrate (red arrow) being unwound by CMG. Right panel shows the fraction of DNA unwound against *Dm*CMG concentration from three independent experiments (mean ± SD). (C and D) *Dm*CMG-mediated unwinding of fork DNA in the (C) absence or (D) presence of clk-SA^Lag^. DNA contained a Cy5 at the 5′ end of the leading-strand template. Right panel shows the percentage of DNA unwound against *Dm*CMG concentration from three independent experiments (mean ± SD). In all gel images, lanes 1–5 correspond to reactions containing 0, 5, 25, 50, and 100 nM *Dm*CMG. Lane 6 corresponds to heat-denatured fork DNA that marks the position of the Cy5-labeled strand. See also Figure S4.

To examine the interaction of isolated CMG with site-specific DPCs, we designed fork DNA templates bearing a clk-SA either on the leading- (clk-SA^Lead^) or lagging-strand (clk-SA^Lag^) templates. Our results with bio-SA blocks suggest that only clk-SA^Lead^ will impair helicase activity. Indeed, *Dm*CMG was unable to unwind templates containing clk-SA^Lead^ (Figures 4A and 4B). It was shown that replication forks can traverse an intact DPC on the leading-strand template (DPC^Lead^) in *Xenopus* egg extracts (Duxin et al., 2014), and two models were proposed to explain this result. One is that the CMG ring transiently opens, as suggested for LTag helicase (Yardimci et al., 2012a), to traverse DPC^Lead^. The alternative model envisages an additional 5′-to-3′ helicase translocating along the lagging-strand template to unwind past DPC^Lead^. We tested the ability of *Dm*CMG to bypass a clk-SA^Lead^ when it is allowed to translocate along DNA for extended periods of time. However, *Dm*CMG failed to efficiently unwind the clk-SA^Lead^-modified DNA even after 120 min of incubation with ATP (Figure S4B), supporting the notion that other factors in egg extracts are required for bypass of a leading-strand protein roadblock (Larsen et al., 2019, Sparks et al., 2019). On the other hand, clk-SA^Lag^ did not inhibit the helicase activity of *Dm*CMG (Figures 4C and 4D), in agreement with our results with DNA containing bio-SA blocks (Figures 1D and 1E).

### Kinetics of CMG Translocation Is Dependent on the Nature of the Lagging-Strand Protein Barrier

To gain further temporal resolution of DNA unwinding by CMG, we sought to inspect the dynamics of CMG translocation by using a fluorescence-based assay. To monitor the kinetics of DNA unwinding in real time, we designed a fork DNA substrate modified with a Cy5 fluorophore at the 5′ end of the leading-strand template and a black hole quencher, BHQ2, at the 3′ end of the lagging-strand template (Figure S5A). The Cy5 signal is quenched due to the presence of a proximal BHQ2 when the DNA is duplexed, and its fluorescence increases upon separation of the two strands (Figure S5A, compare lanes 1 and 3). Similar substrates were used previously to characterize the dynamics of other replicative helicases (Donmez and Patel, 2008, Nandakumar et al., 2015). *Dm*CMG was first allowed to bind the labeled substrate in the presence of ATPγS as before. To achieve single-turnover DNA unwinding, ATP was added together with excess 40-nt poly-T ssDNA that sequesters free helicase. Cy5 fluorescence signal increased with time, which was strictly dependent on the addition of CMG and ATP (Figure S5B), confirming that the fluorescence intensity reflects the DNA unwinding by the helicase. We also confirmed that poly-T oligonucleotide serves as a trap for free helicase (Figure S5C).

**Figure 5:**
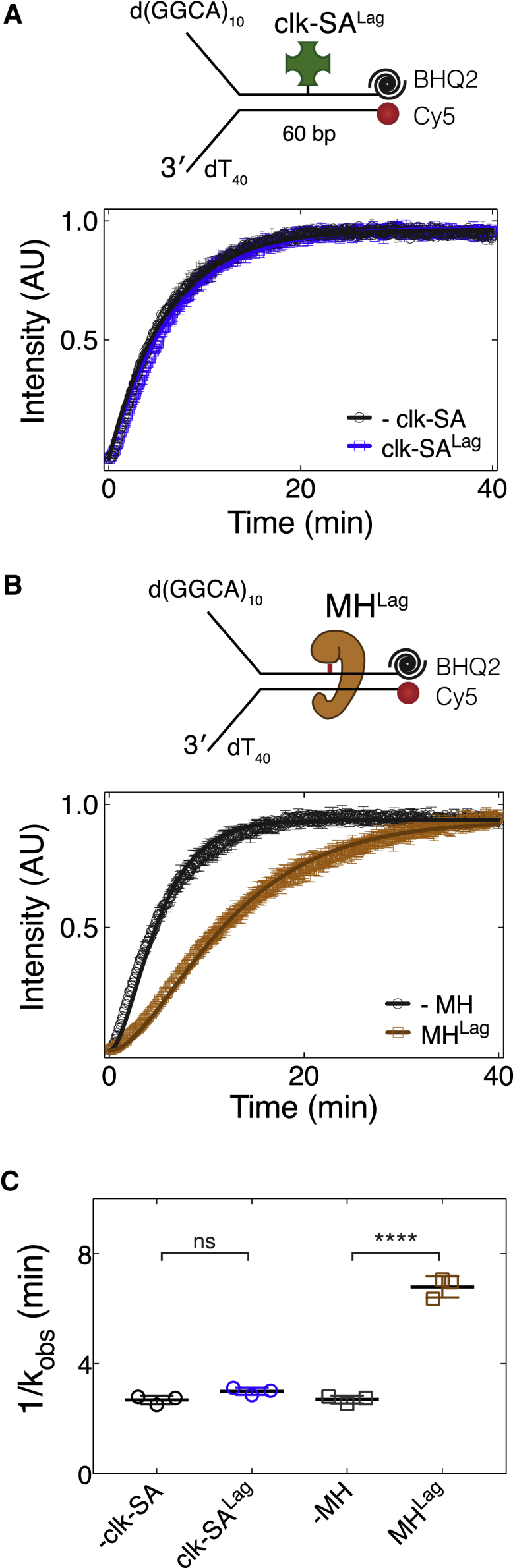
Kinetics of CMG Translocation Is Dependent on the Nature of Lagging-Strand Protein Barrier (A) Single turn-over fluorescence time-course unwinding assays performed using fork DNA substrates with (blue) or without (black) clk-SA^Lag^. (B) Single turn-over fluorescence time-course unwinding of uncrosslinked (black) or MH^Lag^-modified (brown) fork DNA. Fork substrates were labeled with Cy5 fluorophore at the 5′ end of the leading-strand template and contained a BHQ2 fluorescence quencher at the complementary 3′ end. (C) Observed rate constants measured by fitting the data in (A) and (B) to Equation 2 (see the STAR Methods). Data represented here are mean ± SD from three independent experiments. Solid lines in (A) and (B) represent fits to Equation 2. n.s., not significant; ^∗^p < 0.05, ^∗∗^p < 0.01, ^∗∗∗^p < 0.001. See also Figure S5.

To determine whether a protein barrier on the non-translocation strand influences the kinetics of CMG translocation, we repeated the fluorescence assay on DNA bearing a covalently attached lagging-strand SA. *Dm*CMG unwound DNA modified with a clk-SA^Lag^ with the same dynamics as it unwound non-crosslinked substrate (Figures 5A and 5C), indicating that a bulky obstacle on the non-translocating strand does not slow down the helicase. The absence of a significant time delay in bypassing clk-SA^Lag^ makes it unlikely that CMG undergoes a major conformational change, such as opening part of its ring to exclude the lagging-strand template during bypass. If such remodeling of CMG occurs at a lagging-strand roadblock, it neither requires an accessory factor, such as Mcm10, nor causes the helicase to pause, as observed for replication forks in egg extracts (Duxin et al., 2014, Fu et al., 2011).

Because isolated CMG can bypass clk-SA^Lag^ without delay in our assays, we reasoned that the transient fork stalling at lagging-strand protein barriers in *Xenopus* egg extracts must be due to either other components of the eukaryotic replisome interacting with this strand or the type of the protein barrier used in these experiments. Although we conjugated SA to DNA via click chemistry, work in egg extracts involved the use of either M.HpaII methlytransferase (MH) covalently crosslinked to a single fluorinated cytosine (5-fluoro-2′-deoxycytidin, 5FdC) (Duxin et al., 2014) or SA bound to dual biotin-modified DNA (Fu et al., 2011). To test whether MH crosslinked to the lagging-strand template (MH^Lag^) slows down CMG, we made a fork DNA template containing MH^Lag^ located at the center of the duplex region and performed fluorescence-based unwinding assay. Unlike clk-SA^Lag^ (Figure 5A), MH^Lag^ significantly slowed down the kinetics of CMG-mediated unwinding (Figures 5B and 5C, an average delay of 4.09 ± 0.24 min), suggesting that the origin of fork pausing observed in extracts is the interference of helicase translocation by MH^Lag^.

Crystal structures of other methyltransferases trapped on DNA using the same strategy demonstrate that although the enzyme is crosslinked to a base on one strand, it interacts with both DNA strands (Klimasauskas et al., 1994, Kumar et al., 1994). Thus, we hypothesized that MH^Lag^ locks onto and stabilizes duplex DNA, decelerating CMG translocation. To investigate whether MH alters the stability of duplex DNA, we measured heat-induced melting of a short segment of dsDNA. To this end, a Cy5-labeled oligonucleotide was annealed to an oligonucleotide bearing a 5FdC base and conjugated to MH (Figure S5D). Subsequently, an excess of BHQ2-modified competitor oligonucleotide complementary to the Cy5-modifed oligonucleotide was added and reactions were incubated at different temperatures and separated on non-denaturing polyacrylamide gel. The addition of BHQ-labeled oligonucleotide led to fluorescence loss due to melting of duplex and hybridization of Cy5- and BHQ2-labeled oligonucleotides, quenching Cy5 fluorescence (Figure S5D). Although the fluorescence from non-crosslinked DNA was lost with increasing temperature, MH-conjugated duplex was resistant to heat at least up to 60°C, indicating that MH stabilizes dsDNA (Figure S5D). In contrast, clk-SA-modified DNA denatured with the same kinetics as non-crosslinked DNA (Figure S5E). Therefore, we propose that MH^Lag^-induced delay in DNA unwinding by CMG is due to stabilization of duplex DNA rather than steric hindrance of helicase translocation.

To further validate the idea that CMG pausing at MH^Lag^ is due to methyltransferase latching onto and stabilizing dsDNA, we tested whether unfolding MH^Lag^ on the fork DNA substrate used in Figure 5B would speed up CMG-mediated unwinding. Heating MH^Lag^-modified fork DNA led to denaturation of the protein barrier, confirmed by a further shift on polyacrylamide gel (Figure S5F, lane 3). Although a fraction of MH dissociated from DNA, the majority of fork DNA contained unfolded MH^Lag^. Importantly, CMG unwound heat-treated MH^Lag^-modified DNA at a faster rate than it unwound native MH^Lag^-crosslinked substrate (Figures S5G and S5H), strongly suggesting that helicase pausing at MH^Lag^ stems from interactions of the methyltransferase with DNA beyond the crosslinked base.

### Single-Molecule Detection of CMG Pausing at a Lagging-Strand Methyltransferase Block

**Figure 6:**
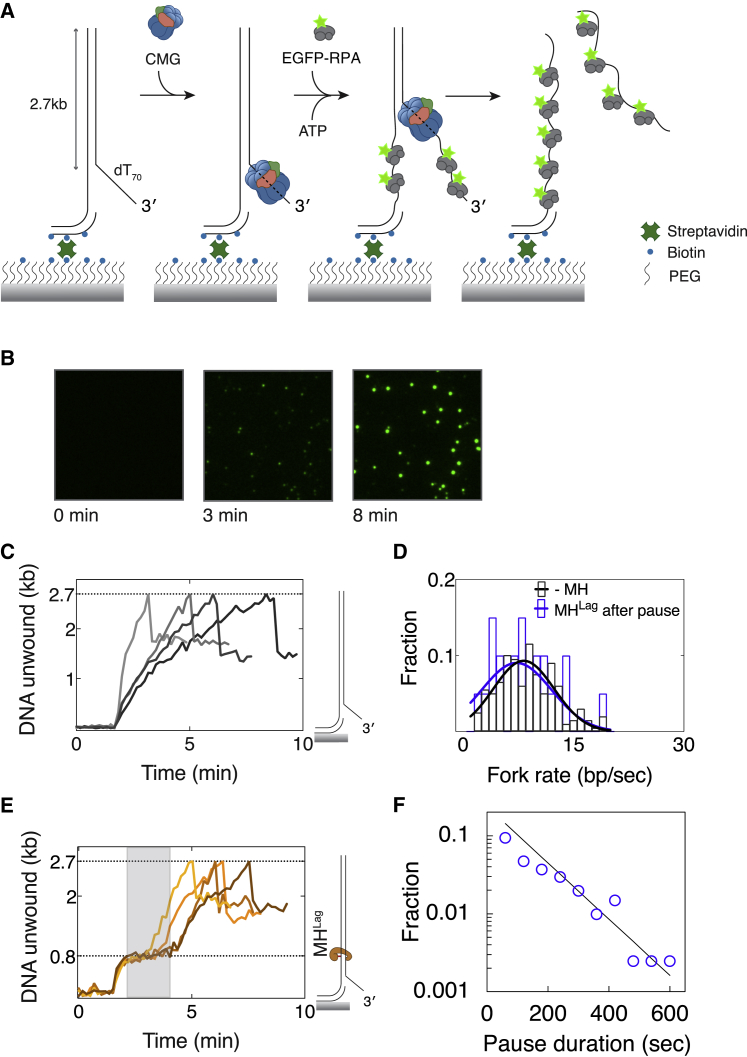
Single-Molecule Detection of CMG Pausing at a Lagging-Strand Methyltransferase Block (A) Schematic representation of experimental approach used in single-molecule DNA unwinding assays. (B) Images of a sample field of view showing accumulation of EGFP-RPA fluorescence signal at different time points from the addition of EGFP-RPA into the chamber. (C) Example unwinding traces of DNA substrates without a protein barrier. Traces exhibit a signal drop upon completion of unwinding due to dissociation of the leading-strand template (depicted in A). (D) Distribution of average fork rates measured in fully unwound substrates without MH (black) and after bypassing MH^Lag^ (blue). Number of molecules are n(-MH) = 199, n(MH^Lag^ after pause) = 20. (E) Sample unwinding traces of DNA substrates modified with MH^Lag^. Pausing observed at 800 bp is highlighted with gray rectangle. (F) Distribution of pause durations observed in molecules exhibiting a pausing event (n = 109). The solid line is a fit to a single exponential. See also Figure S6.

To directly monitor helicase stalling at MH^Lag^, we designed a single-molecule assay in which unwinding of surface-immobilized DNA is measured through binding of fluorescently tagged RPA. Similar assays were previously used to study helicase-catalyzed DNA unwinding at the single-molecule level (Fili et al., 2010). To this end, we generated a 2.7-kb linear substrate bearing multiple biotins at one end for surface attachment and a 3′ dT_70_ ssDNA tail for CMG binding (Figure 6A). After immobilizing DNA on the SA-functionalized surface of a microfluidic flow chamber, *Dm*CMG was introduced in the presence of ATPγS for its binding to the 3′ ssDNA tail. To initiate DNA unwinding and monitor the extent of unwound DNA, EGFP-tagged RPA (EGFP-RPA) was drawn into the flow cell together with ATP (Modesti, 2011). We detected gradual accumulation of EGFP-RPA on many molecules using total internal reflection fluorescence (TIRF) microscopy (Figure 6B). The fluorescence signal increase was dependent on prior CMG assembly, indicating that RPA accumulation occurs as a consequence of CMG-driven DNA unwinding (Figure S6A). Upon unwinding of the entire substrate, release of the strand not coupled to the surface led to a sudden drop of EGFP-RPA intensity on the majority of molecules (Figure 6C). Under our experimental conditions, CMG unwound dsDNA at an average speed of 8.2 ± 4.2 bp/s (Figure 6D, black), comparable to *in vitro* fork rates with the minimal eukaryotic replisome (Lewis et al., 2017, Yeeles et al., 2017) and translocation speed of CMG on ssDNA (Wasserman et al., 2018). To observe MH^Lag^-induced helicase pausing, we introduced MH^Lag^ approximately 800 bp away from the 3′ ssDNA tail. A total of 26% of molecules exhibited a discernible pausing event (more than 30 s) near the position of MH^Lag^ (Figure 6E). The distribution of pause durations indicates the helicase pauses an average period of 4.63 ± 0.22 min at MH^Lag^ (Figure 6F), paralleling the time delay seen in our ensemble assays (Figure 5B). After traversal of the barrier, average unwinding rate becomes indistinguishable from that on unadducted substrate (Figure 6D). In addition, we designed a DNA substrate containing an MH^Lead^ 800 bp away from the fork junction to detect CMG arrest at the protein barrier (Figure S6B). Concomitantly, on 80% of the molecules, fluorescence intensity reached a plateau closely matching the average intensity at which unwinding paused on MH^Lag^-modified substrate (Figures S6C and S6D). Together, single-molecule visualization of DNA unwinding by CMG reveals that although helicase indefinitely stalls upon encountering MH^Lead^, it transiently pauses at MH^Lag^, likely due to increased stability of duplex DNA, and resumes its normal translocation speed once the barrier is bypassed.

### Replication Fork Dynamics at Different DPCs in *Xenopus* Egg Extracts

**Figure 7:**
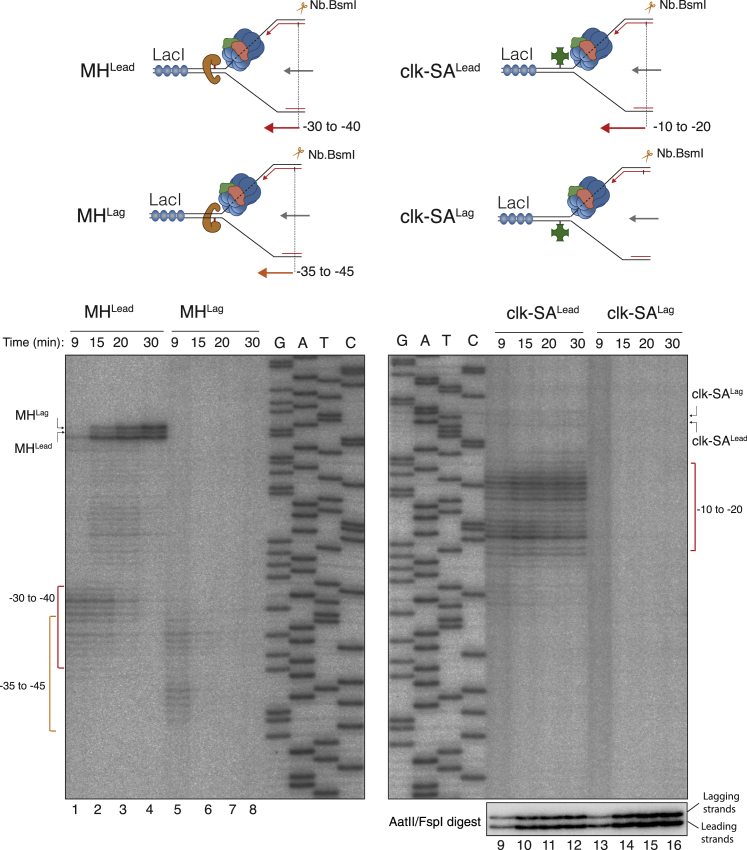
Replication Fork Dynamics at Different DPCs in *Xenopus* Egg Extracts Plasmids modified with MH^Lead^, MH^Lag^, clk-SA^Lead^, and clk-SA^Lag^ were replicated in egg extracts in the presence of LacI to ensure that a single fork encounters the DPC (Duxin et al., 2014). At the indicated time points, samples were digested with Nb.BsmI and analyzed on a denaturing polyacrylamide gel. The upper schematics depict the nascent leading-strand products liberated by Nb.BsmI digest. After replication, plasmids containing clk-SA^Lead^ and clk-SA^Lag^ were also digested with AatII and FspI (bottom radiograph) that cleave on either side of the DPC and allows to monitor the nascent leading- and lagging-strand extensions past the DPC. Note that ∼50% of the plasmid contained crosslinked SA (Figure S7).

Slowed unwinding seen with isolated CMG on MH^Lag^-conjugated substrate (Figures 5B and 6E) recapitulates transient fork stalling in *Xenopus* egg extracts observed with the same roadblock. These results suggest that replisome pausing in extracts is specifically caused by the stabilization of duplex DNA by MH^Lag^ and not because CMG needs to be remodeled to bypass a bulky barrier on the non-tracking strand. If this is true, clk-SA^Lag^ should not stall the replisome, as this modification does not affect CMG helicase activity (Figure 5A). To test this idea, we generated plasmids modified with either SA crosslinked by click chemistry (Figure S7) or covalently trapped MH. These plasmids also contained 48 repeats of the lacO sequence near the protein crosslink. When replicated in egg extracts in the presence of lac repressor protein, LacI, the rightward replication fork stalls at the array while the leftward fork encounters the protein crosslink on either the leading- or lagging-strand templates (Figure 7, upper schemes) (Dewar et al., 2015, Duxin et al., 2014). To address the impact of the protein block on replisome progression, nascent leading strands were analyzed on a denaturing polyacrylamide gel following Nb.BsmI digest. As previously described, in the presence of MH^Lead^, nascent leading strands first stalled ∼30 to 40 nt upstream of the adduct for ∼10–15 min (Figure 7, lanes 1–4), consistent with the footprint of CMG, which is stalled at the DPC (Duxin et al., 2014). When replication forks encountered clk-SA^Lead^, nascent leading strands also stalled but at a position closer to the crosslinked site compared to MH^Lead^ and persisted for a prolonged period of time ∼10–20 nt upstream of the DPC (Figure 7, lanes 9–12). Because the linker used to crosslink SA spans a distance of ∼15 nt, we reason that the nascent leading strand can be extended closer to the crosslinked base most probably because the CMG helicase can encircle and extend the linker into its central channel until it reaches SA. We next investigated the lagging-strand blocks. MH^Lag^ led to transient but clearly observable stalling of nascent leading strands ∼35–45 nt from the adduct (Figure 7, lane 5; Duxin et al., 2014); however, absolutely no stalling was detected in the presence clk-SA^Lag^ (Figure 7, lanes 13 and14), although nascent extension products clearly accumulated between 9 and 15 min (Figure 7, lanes 13 and 14, bottom radiograph). These data corroborate our observations with purified CMG and indicate that in the context of a full replisome, CMG does not encircle the displaced strand and, consequently, ring opening is not necessary to traverse a protein barrier that exclusively interacts with the lagging-strand template.

## Discussion

Here, we investigated the impact of various protein-DNA complexes on the activity of the eukaryotic replicative helicase CMG while unwinding the DNA double helix at the replication fork. To address whether any domain of CMG encircles duplex DNA within the central pore while it advances on DNA, we studied the ability of CMG to unwind DNA templates modified with site-specific roadblocks. Using model fork DNA templates, we observed that *Drosophila* and yeast CMG arrest at a bio-SA block on the leading-strand template while bypassing a lagging-strand bio-SA complex without removing SA. Consistently, we showed that covalently coupled SA on the leading- but not the lagging-strand template blocks the helicase. Thus, our results agree with the steric exclusion model.

Similar fork unwinding assays by others using *Sc*CMG showed helicase inhibition by lagging-strand protein blocks (Langston and O’Donnell, 2017, Langston et al., 2017). Together with structural studies demonstrating duplex DNA within the N-tier of MCM (Georgescu et al., 2017), it was proposed that after entering into the MCM ring, the lagging-strand template either bends back to exit from the same pore or curves to escape through a gap between MCM zinc fingers (Langston and O’Donnell, 2017, Langston et al., 2017, O’Donnell and Li, 2018). In line with this model, it was found that *Sc*CMG is able to displace a single SA from the lagging-strand template to some extent (Langston and O’Donnell, 2017). However, given that the stability of SA is relatively low on dsDNA modified with an internal biotin-dT (Figure S1B), a significant portion of SA may have released in a CMG-independent manner. We observed helicase inhibition when TA was bound to biotin on the lagging-strand template with a short linker, consistent with results obtained with *Sc*CMG reported previously (Langston and O’Donnell, 2017, Langston et al., 2017). Importantly, when CMG was bound to the fork DNA before TA, no inhibition was observed, indicating that CMG binding but not translocation is disrupted by the presence of a protein block in these assays. It is likely that the presence of the PEG4 linker between biotin and the thymidine base in our original fork DNA templates relieved the obstruction of CMG binding to SA-bound DNA because this substrate was efficiently unwound by CMG. We conclude that the helicase activity of neither *Drosophila* nor yeast CMG is impaired when a lagging-strand protein barrier is encountered during translocation. Thus, if the yeast CMG structure in which the MCM N-tier encircles duplex DNA at the fork junction (Georgescu et al., 2017) reflects the DNA-unwinding state of the helicase, there must be a gap in this region wide enough to allow the protein-bound lagging-strand template to freely escape from the central channel of the helicase.

In *Xenopus* egg extracts, replication forks transiently stall upon encountering a dual bio-SA complex (Fu et al., 2011) or a covalent methyltransferase (Duxin et al., 2014) on the excluded strand. The origin of the transient fork arrest at a lagging-strand roadblock in egg extracts has been unclear. It was thought that the interaction of CMG with the excluded strand through the outer surface of the helicase might be causing the helicase to stall (Fu et al., 2011). In support of this idea, wrapping of the excluded strand on the exterior of replicative helicases has been reported (Carney and Trakselis, 2016, Carney et al., 2017, Graham et al., 2011). An alternative explanation for this stalling was that the fork junction is buried inside the helicase central pore and that slight opening of the MCM ring can facilitate bypass (Langston and O’Donnell, 2017). In addition, Mcm10 was found to aid CMG in bypassing a lagging-strand block (Langston et al., 2017), implying that Mcm10 in egg extracts may associate with the helicase to traverse the barrier. This model seems plausible given that Mcm10 plays a role in the eviction of the excluded strand during initiation (Douglas et al., 2018). However, as CMG does not require ancillary factors to bypass a covalent lagging-strand roadblock (Figures 4D and 5), it is unlikely that Mcm10 is needed to open the Mcm2-7 ring for bypass of lagging-strand protein barriers. The absence of significant stalling in the presence of clk-SA^Lag^ suggests that a bulky lesion on the lagging-strand template does not influence CMG translocation. Thus, even if CMG interacts with the excluded strand, a protein bound to this strand is not sufficient to cause detectable helicase pausing. Importantly, MH^Lag^ that caused brief fork stalling in extracts also delayed DNA unwinding by isolated CMG. In addition, clk-SA^Lag^ did not lead to any detectable fork stalling in extracts. The remarkable correlation between the dynamics of isolated CMG and fork progression in egg extracts at site-specific protein barriers implies that MH^Lag^-induced fork stalling in extracts is due to the ability of this enzyme to stabilize dsDNA rather than acting as a steric barrier. Thus, we envisage that a protein that exclusively interacts with the displaced strand does not influence CMG progression during replication. In agreement with this, two converging CMG complexes bypass one another without stalling in egg extracts during replication termination (Dewar et al., 2015).

Reported eukaryotic replication fork rates differ significantly among organisms. For example, forks progress with an average speed of 1–2 kb min^−1^ in human cell lines (Conti et al., 2007) and 0.3–0.5 kb min^−1^ in *Xenopus* egg extracts (Loveland et al., 2012, Mahbubani et al., 1992, Yardimci et al., 2010). Transient stalling at duplex stabilizing assemblies implies that fork progression is often interrupted during cellular replication by DNA-binding factors. As histones are probably the most abundant proteins on DNA, the replisome accommodates histone chaperones, such as FACT, to promptly overcome nucleosomes (Foltman et al., 2013, Gambus et al., 2006, Kurat et al., 2017, Yang et al., 2016). However, when the replisome encounters an aberrant roadblock, such as a methyltransferase-bound site, its progression is hindered. Consistently, replication forks slowly advance through LacI-bound lacO sites in egg extracts (Dewar et al., 2015). Therefore, the overall slower fork rates in egg extracts could be due to the presence of high amounts of DNA-binding factors and the heavily chromatinized state of the DNA.

Dimers of inactive Mcm2-7 complexes are organized such that their N-terminal domains strongly interact with each other and both rings encircle dsDNA at origins of replication (Abid Ali et al., 2017, Bell and Labib, 2016, Evrin et al., 2009, Li et al., 2015, Noguchi et al., 2017, Remus et al., 2009). As evidenced by the independent action of replisomes (Yardimci et al., 2010) and the absence of CMG dimers in *Xenopus* egg extracts (Gambus et al., 2011), the two hexamers most likely split during initiation. Recent results with the reconstituted yeast system indicate that separation of the double hexamer occurs upon CMG formation (Douglas et al., 2018). Once CMG initiates the unwinding of DNA, the N-terminal face of Mcm2-7 advances in front, indicating that sister helicases must bypass one another at the origin of replication (Douglas et al., 2018, Georgescu et al., 2017). As proteins that engage solely with the excluded strand do not slow down CMG, we envision that activated helicases bypass one another without stalling during replication initiation, as seen in termination (Dewar et al., 2015). In the future, it will be important to determine whether a well-defined path exists for the excluded strand with respect to CMG during unwinding.

## STAR★Methods

### Key Resources Table

**Table:** 

REAGENT or RESOURCE	SOURCE	IDENTIFIER
**Bacterial and Virus Strains**		

Max Efficiency DH10Bac competent cells	ThermoFisher	10361012
Rosetta (DE3)pLysS competent cells	Novagen	70956
DH5α	ThermoFisher	12297016

**Chemicals, Peptides, and Recombinant Proteins**		

Peptide (DYKDDDDK)	Peptide Chemistry, STP, The Francis Crick Institute	N/A
Gamma-ATP [γ-^32^P], Easy Tide	Perkin Elmer	BLU502A100UC
EZ link-NHS-PEG4-Biotin	Thermo Fisher	21330
Streptavidin from *Streptomyces avidinii*	Sigma-Aldrich	S4762
Microspin G50 Spin columns	GE Healthcare	GE27-5330-01
3.5 kDa MWCO Dialysis Membrane	Fisher Scientific	11425859
12-14 kDa MWCO Dialysis Membrane	Fisher Scientific	11475849
3 kDa MWCO Vivaspin concentrator	Generon	VS0192
DMSO (Dimethyl sulfoxide), anhydrous	Sigma Aldrich	276855
DBCO-Sulfo-NHS-Ester	Jena Biosciences	CLK-A124
DBCO-PEG4-NHS-Ester	Jena Biosciences	CLK-A134
Azide-PEG3-Biotin	Jena Biosciences	CLK-AZ104P4
Acrylamide: Bis-Acrylamide 37.5:1	Fisher Scientific	10376643
Agarose	Denville Scientific	CA3510-8
PBS, pH = 7.4	GIBCO	70011044
Sf-900TM III SFM insect cell medium	Thermo Fisher	12658019
Anti-Flag M2 Affinity Gel	Sigma Aldrich	A2220
Ni-NTA agarose beads solution	QIAGEN	30210
EDTA-Free PI tablets	Roche	5056489001
Mini GebaFlex Tube-Dialysis (0.2 ml)	Generon	D070-6-10
NuPAGE 4-12% Bis-Tris Protein Gels	Thermo Fisher	NP0323BOX
ATPγS (Adenosine 5′-0-(3-thiotriphosphate))	Roche	11162306001
ATP	Roche	11140965001
Dynabeads M-280 Streptavidin	Invitrogen	11205D
Biotin	Sigma Aldrich	B4501
Amphotericin B (Fungizone)	GIBCO	15290018
Gentamycin	GIBCO	15750060
Ampicillin	Roche/Sigma	10835269001
Benzonase nuclease	Sigma	E1014
HpaII Methyltranferase	NEB	M0214
T4 PNK	NEB	M0201
T4 Ligase	NEB	M0202
Nt.BbvCl	NEB	R0632
Nb.BsmI	NEB	R0706
HpaII Methyltransferase (M.HpaII)	NEB	M0214
Nunc 384 shallow well plate, black	Thermo Fisher	264705

**Critical Commercial Assays**		

QIAGEN Gel Extraction Kit	QIAGEN	28704
QIAGEN PCR Purification Kit	QIAGEN	28104
QIAGEN Miniprep Kit	QIAGEN	27104
HiTrap SPFF column (1ml)	GE Healthcare	17-5054-01
MonoQ 5/50 GL column (1ml)	GE Healthcare	17516601
MonoQ PC 1.6/5 GL column (0.1ml)	GE Healthcare	17067101
Superdex 200 10/300 GL column (24 ml)	GE Healthcare	28990944

**Experimental Models: Cell Lines**		

Spodoptera frugiperda (SF9) cells	Cell Services, STP, The Francis Crick Institute	N/A
Spodoptera frugiperda (Sf21) cells	Structural Biology, STP, The Francis Crick Institute	N/A
Hi Five cells	Cell Services, STP, The Francis Crick Institute	N/A

**Experimental Models: Organisms/Strains**		

*Xenopus laevis* (females)	Nasco	LM0053MX
*Xenopus laevis* (males)	Nasco	LM00715MX
*S. cerevisiae*: yJCZ3, Strain background: W303	Zhou et al., 2017	N/A

**Oligonucleotides**		

Oligonucleotides used in all DNA templates (See Table S2)	Integrated DNA Technologies	N/A

**Recombinant DNA**		

pFB1-Mcm2	Ilves et al., 2010	N/A
pFB1-Mcm3	Ilves et al., 2010	N/A
pFB1-Mcm4	Ilves et al., 2010	N/A
pFB1-Mcm5	Ilves et al., 2010	N/A
pFB1-Mcm6	Ilves et al., 2010	N/A
pFB1-Mcm7	Ilves et al., 2010	N/A
pFB1-Psf1	Ilves et al., 2010	N/A
pFB1-Psf2	Ilves et al., 2010	N/A
pFB1-Psf3	Ilves et al., 2010	N/A
pFB1-Sld5	Ilves et al., 2010	N/A
pFB1-Cdc45	Ilves et al., 2010	N/A
Plasmid-EGFP-RPA	Modesti, 2011	N/A
pHY39	This work	N/A

**Software and Algorithms**		

GraphPad Prism7	https://www.graphpad.com/scientific-software/prism/	RRID:SCR_002798
ImageJ	https://imagej.nih.gov/ij/	RRID:SCR_003070
FUJIFILM-FLA-5000	FUJIFILM	N/A
Typhoon-FLA-9500	GE Healthcare	N/A
PHERAStar	https://www.bmglabtech.com/pherastar-fsx/	N/A
Adobe Illustrator	https://www.adobe.com/products/illustrator.html	RRID:SCR_014198

### Contact for Reagent and Resource Sharing

Further information and requests for reagents should be directed to and will be fulfilled by the Lead Contact, Hasan Yardimci (Hasan.Yardimci@crick.ac.uk).

### Experimental Model and Subject Details

#### Xenopus laevis

Egg extracts were prepared using *Xenopus laevis* (Nasco Cat #LM0053MX, LM00715MX). All experiments involving animals were approved by the Danish Animal Experiments Inspectorate, and conform to relevant regulatory standards and European guidelines.

#### Insect Cells

Sf9 insect cells (Thermo Fisher) were used to generate baculoviruses and High Five insect cells (Thermo Fisher) were used for *Dm*CMG expression.

#### Bacteria Strains

*E.coli* strains Rosetta(DE3)pLysS (Novagen), DH10Bac (Thermo Fisher), and DH5α (Thermo Fisher) were used for EGFP-RPA expression, bacmid generation, and plasmid cloning, respectively.

#### Yeast Strains

yJCZ3 yeast strain (background, W303) was used for *Sc*CMG expression.

### Method Details

#### DNA Substrates

We designed a variety of DNA substrates containing different modifications. Sequences of oligonucleotides (oligos) used in each substrate can be found in Table S1. DNA substrates were made using a combination of oligos as indicated in Table S2.

In general, fork DNA substrates were made by mixing equimolar amounts (10 μM final) of oligos in STE buffer (10 mM Tris-HCl pH 8.0, 100 mM NaCl, 1 mM EDTA), heating to 85°C and subsequently allowing to slowly cool down to room temperature (RT). When necessary, resulting nicks were sealed by ligating with T4 DNA ligase (NEB).

To prepare DNA templates containing site-specific biotin modification with PEG4 linker, approximately 200 μg of amine-modified DNA was mixed with 2 mg of EZ-link-NHS-PEG4-Biotin (ThermoFisher) in phosphate buffered saline (PBS) and incubated for 16 hours at room temperature (RT). DNA was separated on either 3% agarose gel or 8% polyacrylamide gel (PAGE), and the resulting substrate was purified using either a gel extraction kit (QIAGEN) or electroelution, respectively. Electroelution was performed using 3.5 kDa MWCO dialysis membrane (Spectra/Por, Spectrum Labs) in 1x TBE buffer. Where indicated, fork DNA substrates were labeled with [γ-^32^P]-ATP using T4 PNK enzyme (NEB). Radiolabelled samples were spun through a MicroSpin G50 column (GE Healthcare) equilibriated with TN buffer (10 mM Tris-HCl, pH 8.0, 20 mM NaCl) to remove excess radionucleotide. To selectively purify biotin-modified DNA, 1 mg of streptavidin (SA), was mixed with 2 μg of DNA substrate and incubated for 3 hours. After separating on 8% PAGE, bands corresponding to SA-bound DNA were excised and purified by electroelution. When desired, DNA samples were concentrated with 0.5ml VivaSpin (3 kDa MWCO) concentrator (Generon).

To generate DNA templates containing site-specific biotin-dT modification, oligos were annealed and purified as described above. 1 mg of traptavidin (TA) was mixed with 2 μg of DNA substrate and incubated for 3 hours for TA binding. To purify substrates with stably bound TA, TA-binding reaction was mixed with biotin (80 μM final) and incubated at 37°C for 10 min. After separating on 8% PAGE, bands corresponding to TA-bound DNA were excised, purified, and concentrated as before.

To functionalize SA with DBCO, 500 μL of 20 mg/ml SA in PBS was mixed with 60 μL of DBCO-Sulfo-NHS-Ester (Jena Biosciences, 100 mM dissolved in DMSO) and incubated at RT for 3 hours, rotating. DBCO-SA was dialyzed against PBS using 12- 14 kDa MWCO membrane (Spectra/Por, Spectrum Labs) to remove excess DBCO.

To prepare fork DNA substrates containing site-specific DPCs, oligos were annealed and ligated. After separating the resulting azide- or 5FdC-modified fork DNA on 8% PAGE, they were purified by electroelution as described above. The products were dialyzed against TN buffer and further concentrated. Azide-modified fork DNA templates were mixed with DBCO-modified SA (7 mg/ml final) and incubated at 37°C for 3 hours. 5FdC-modified fork templates were mixed with HpaII methyltransferase (M.HpaII) in 1:4 DNA to protein molar ratio in methyltransferase buffer (50 mM Tris-HCl, pH 7.5, 0.5 mM 2-mercaptoethanol (β-ME), 10 mM EDTA, NEB) supplemented with 100 μM S-adenosylmethionine (NEB), and incubated at 37°C for 3 hours. The reactions were run on 3% agarose gel to separate DPC-conjugated DNA substrate from unconjugated DNA as well as excess DBCO-SA and MHpaII. The bands corresponding to DPC-modified and unmodified DNA substrates were excised, separately isolated through electroelution, and concentrated.

DNA substrates used in single-molecule experiments were constructed by ligating a 2.7kb linear duplex to a biotinylated ‘handle’. First, pHY10 (Duxin et al., 2014) was modified via site-directed mutagenesis to introduce a KpnI site overlapping the M.HpaII-recognition sequence to generate pHY39. 2.7 kb fragment from this vector was PCR amplified either with Oligo-18 and Oligo-19 for MH^Lag^ or with Oligo-20 and Oligo-21 for MH^Lead^, and digested with Nt.BbvCl (NEB). After nicking, Oligo-22 and Oligo-23 (10 μM final) were added, and the reaction was heated to 50°C for 10 min to capture displaced strands. DNA was then gel purified and ligated to Oligo-Fluo-1. M.HpaII conjugation was performed as before. To eliminate substrates lacking MHpaII, DNA was digested with KpnI (NEB), separated on a 1% agarose gel, and purified by electroelution.

To prepare the biotinylated handle, 568 bp fragment of pUC19 plasmid was PCR amplified using Oligo-12 and Oligo-13 in the presence of biotin-16-dUTP (Roche), digested with Nt.BbvCl, and gel purified. A ‘spacer’ was prepared by annealing Oligo-15, Oligo-16, and Oligo-17 containing 3′ dT_70_ ssDNA tail for CMG assembly. While one end of the spacer contains an overhang complementary to the Nt.BbvCI-treated biotin handle, the other end has an overhang complementary to one end of the nicked 2.7 kb duplex. The spacer was first ligated to nicked biotin-modifed PCR product with T4 Ligase (NEB), and gel purified. The resulting construct was then ligated to MH-modified 2.7 kb DNA, separated on 0.8% agarose, and purified by electroelution.

To prepare MH-conjugated plasmids for replication assays, pJLS2 (Larsen et al., 2019) was digested either with Nt.BbvCI or Nb.BbvCI (NEB), and ligated to Oligo-Fluo-2 or Oligo-Fluo-3, respectively. The ligated products were gel purified, and crosslinked with M.HpaII as previously described (Duxin et al., 2014).

To prepare clk-SA modified plasmids pJLS2 was digested with BbvCI, and 5′-TCAGCAGGTCCGGCTTAAGCCTTATAAAGGTACC-3′ sequence was inserted to generate pNBL101. pNBL101 was digested either with Nt.BbvCl or Nb.BbvCl, and ligated to Oligo-azide-3 or Oligo-azide-4, respectively. Ligated products were gel purified. To functionalize SA with DBCO-PEG4, 500 μl of 20 mg/ml SA in PBS was mixed with 60 μl of DBCO-PEG4-NHS-Ester (Jena Biosciences, 100 mM dissolved in DMSO) and incubated at RT for 3 hours, rotating. DBCO-PEG4-SA was dialyzed against PBS using 12-14 kDa MWCO membrane (Spectra/Por, Spectrum Labs) to remove excess DBCO-PEG4. DBCO-PEG4-modified SA (15 mg/ml final) was mixed with azide-modified plasmids (50 ng/μl final) and incubated at 37°C for 3 hours for click reaction.

#### Expression and Purification of Recombinant DmCMG

*Dm*CMG was expressed and purified as described before (Abid Ali et al., 2016, Ilves et al., 2010) with minor changes. Briefly, following bacmid generation for each subunit of *Dm*CMG, Sf21 cells were used for the initial transfection and subsequent virus amplification stages to generate P2 stocks using serum-free Sf-900TM III SFM insect cell medium (Invitrogen/GIBCO). In the P3 virus amplification stage, 100 mL Sf9 cell (0.5x10^5^/ml) cultures were infected with 0.5 mL of P2 stocks with an approximate MOI of 0.1 for each virus, and incubated in 500 mL Erlenmeyer sterile flasks (Corning) for 4 days at 27°C, shaking at 100 rpm. 4L of Hi-Five cells (10^6^/ml) supplemented with 10% FCS were infected using fresh P3 stocks with MOI of 5. Cells were incubated at 27°C and harvested after 60 hours. Cell pellets were first washed with PBS supplemented with 5 mM MgCl_2_, resuspended (50 mL buffer per 1 L of Hi-Five cell culture) in resuspension buffer (25 mM HEPES pH 7.5, 1 mM EDTA, 1 mM EGTA, 0.02% Tween-20, 10% glycerol, 15 mM KCl, 2 mM MgCl_2_, 2 mM β-ME, PI tablets), and frozen in 10 mL aliquots in dry ice.

All steps during purification were performed at 4°C unless otherwise specified. Cell pellets were thawed and lysed by applying at least 50 strokes per 30 mL of cell pellets using tissue grinders (Wheaton, 40 mL Dounce Tissue Grinder). Lysed cells were centrifuged at 24,000 g for 10 min and cell debris was removed. The supernatant was incubated with 2 mL M2 agarose flag beads (Sigma Aldrich) equilibrated with Buffer C (25 mM HEPES pH 7.5, 1 mM EDTA, 1 mM EGTA, 0.02% Tween-20, 10% glycerol, 1 mM DTT) for 2.5 hours. Supernatant was removed by centrifugation at 200 g for 5 min and the flag beads were washed with 30 mL of Buffer C-100 (25 mM HEPES pH 7.5, 100 mM KCl, 1 mM EDTA, 1 mM EGTA, 0.02% Tween-20, 10% glycerol, 1 mM DTT). Beads were incubated with Buffer C-100 supplemented with 200 μg/ml peptide (DYKDDDDK) at room temperature for 15 min to elute bound proteins. The eluate was passed through 1 mL HiTrap SPFF column (GE Healthcare) equilibrated with Buffer C- 100. *Dm*CMG complex was separated with 100-550 mM KCl gradient using 5/50GL MonoQ column (GE Healthcare) in 20 ml. Fractions where *Dm*CMG was eluted included ∼400-450 mM KCl. To further concentrate the sample, pooled fractions were diluted to a final concentration of 150 mM KCl, and loaded onto MonoQ PC 1.6/5 GL (GE Healthcare) column equilibrated with Buffer C-150-NT (25 mM HEPES pH 7.5, 150 mM KCl, 1 mM EDTA, 1 mM EGTA, 10% glycerol, 1 mM DTT). 150-550 mM KCl gradient was applied to the column, and peak fractions were pooled and dialyzed against CMG dialysis buffer (25 mM HEPES pH 7.5, 50 mM sodium acetate, 10 mM magnesium acetate, 10% glycerol, 1mM DTT) using 8 kDa MWCO dialysis tubing (Generon) for 2 hours. The aliquots were flash frozen using liquid nitrogen and kept in −80°C.

#### Expression and Purification of Recombinant ScCMG

The expression and purification of *Sc*CMG were performed as described in Zhou et al., (2017) using yJCZ3 yeast strain with minor changes in the purification step. Briefly, cell cultures were induced with 2% galactose for 3 hours at 30°C, harvested and frozen dropwise in liquid nitrogen. Pellets were ground into powder using a grinder mill. The powder was resuspended (20 mL buffer per 1L cell pellet) in Sc-Res buffer (25 mM HEPES pH 7.5, 1 mM EDTA, 1 mM EGTA, 10% glycerol, 150 mM KCl, 1 mM DTT) supplemented with 25 units/ml benzonase nuclease (Sigma Aldrich) and EDTA-free PI tablets (Roche), and stirred for 30 min at 4°C. Cell debris was removed by centrifugation at 235,000 g. Next, the supernatant was incubated with 4 mL M2 agarose flag beads (Sigma Aldrich) equilibrated with Buffer C for 2.5 hours. After removing the supernatant by centrifuging at 200 g for 5 min and washing beads with 50 mL Buffer C-100, *Sc*CMG was eluted by incubating the beads with C-100 buffer supplemented with 200 μg/ml peptide (DYKDDDDK) for 15 min at room temperature. The eluate was passed through HiTrap SPFF column (GE Healthcare) equilibrated with C-100 Buffer. *Sc*CMG was separated with 100-550 mM KCl gradient using 5/50GL MonoQ column (GE Healthcare) in 20 ml. *Sc*CMG-containing fractions were combined and further purified using Superdex 200 10/300GL gel filtration column (GE Healthcare) equilibrated with C-150-NT buffer (25 mM HEPES pH 7.5, 1 mM EDTA, 1 mM EGTA, 10% glycerol, 150 mM KCl, 1 mM DTT). To concentrate the sample, fractions containing *Sc*CMG were pooled and loaded onto MonoQ PC 1.6/5 GL (GE Healthcare) column, and KCl gradient (150-550 mM) was applied in 2 ml. Peak fractions were pooled and dialyzed against CMG dialysis buffer using 8 kDa MWCO dialysis tubing (Generon) for 2 hours, and the aliquots were flash frozen in liquid nitrogen and kept in −80°C.

#### Expression and Purification of EGFP-RPA

The plasmid for EGFP-RPA expression was obtained from Maura Modesti. Expression and purification of EGFP-RPA were performed as described in Modesti, (2011). Rosetta/pLysS competent cells were used as a host to express the protein. Cells were grown in LB media supplemented with ampicillin and chloramphenicol at 37°C. At exponential phase (OD ∼0.5), expression was induced with 1 mM IPTG, incubator temperature was reduced to 15°C, and cells were incubated further for 20 hours. Cells were harvested by centrifugation at 3500 g for 30 mins, and the cell pellet was washed with PBS.

To purify EGFP-RPA, cell pellets were thawed in lukewarm water, and resuspended in RPA Lysis buffer-2 (40 mM Tris-HCl pH 7.5, 1 M NaCl, 20% glycerol, 4 mM β-ME, 10 mM imidazole) supplemented with EDTA-free PI tablets. Resuspended pellet was sonicated (3 s on, 10 s off, 20 cycles), and cell debris was removed by centrifugation at 20000 g for 1 hour. Supernatant was filtered using 0.45 μm syringe filters (Millipore), and loaded onto 1ml HisTrap FF (GE Healthcare) column equilibrated with RPA-Lysis buffer-1 (20 mM Tris-HCl pH 7.5, 500 mM NaCl, 2 mM β-ME, 20% glycerol, 10 mM imidazole, 1 mM DTT). Linear gradient between 10-300 mM imidazole was applied to separate EGFP-RPA. Fractions containing EGFP-RPA were pooled, and dialyzed against RPA-Dialysis buffer-1 (20 mM Tris-HCl pH 7.5, 50 mM KCl, 0.5 mM EDTA, 10% glycerol, 1mM DTT) using 3.5 kDa MWCO dialysis tubing (Generon) overnight. Next day, the sample was loaded onto HiTrap Heparin column (GE Healthcare) equilibrated with RPA-Dialysis buffer-1. EGFP-RPA was eluted by applying 50-500 mM KCl gradient. Fractions containing EGFP-RPA were pooled, and dialyzed against RPA-Dialysis buffer-2 (20 mM Tris-HCl pH 7.5, 50 mM KCl, 0.5 mM EDTA, 25% glycerol, 1 mM DTT) using 3.5kDa MWCO dialysis tubing (Generon) for 2 hours. EGFP-RPA was aliquoted, flash frozen using liquid nitrogen, and stored in −80°C.

#### Gel-based DNA Unwinding Assays

To attach SA, biotin-modified radiolabelled DNA was incubated either with buffer or excess SA (1 mg/ml final) for 1 hour at RT prior to performing the unwinding assays. 3 nM of DNA substrate in 6 μL reaction was incubated with reported amounts of *Dm*CMG or *Sc*CMG in CMG-binding buffer (25 mM HEPES, pH 7.5, 5 mM NaCl, 10 mM magnesium acetate, 5 mM DTT, 0.1 mg/ml BSA) supplemented with 0.1 mM ATPγS at 37°C for 2 hours. 6 μL of ATP mix (25 mM HEPES pH 7.5, 5 mM NaCl, 10 mM magnesium acetate, 5 mM DTT, 0.1 mg/ml BSA, 5 mM ATP) was added to initiate unwinding and samples were incubated at 30°C for further 10 min. Reactions were terminated with stop buffer containing 0.5% SDS and 20 mM EDTA. To prevent aggregation of CMG-bound DNA that results in some DNA being stuck in the well, we added 5 μM of 40-nt poly-T oligo (Oligo-5) as a competitor DNA together with stop buffer into the reaction. DNA fragments were separated on 12% PAGE in 1x TBE. Gels were mounted on Whatman paper, exposed to storage phosphor screen overnight, and scanned the following day (Typhoon-FLA-9500). For unwinding of Cy5- modified DNA substrates containing clk-SA^Lead^ or clk-SA^Lag^, 5 nM of DNA substrate in a 6 μL reaction was incubated with reported amounts of *Dm*CMG. We applied the same buffer and incubation conditions described for DNA bearing biotin-SA blocks. DNA was separated on 8% PAGE with 1x TBE and imaged on Fujifilm SLA-5000 scanner using 635-nm laser and Fujifilm LPR/R665 filter. ImageJ was used to linearize gel images and quantify the intensity of each band visible on gels.

#### Fluorescence-based Time Course DNA Unwinding Assays

5 nM of Cy5-BHQ2 labeled fork DNA substrate was mixed with 60 nM *Dm*CMG in 30 μL of total reaction volume in CMG binding buffer supplemented with 0.1 mM ATPγS and incubated at 37°C for 2 hours. In the meantime, microplate wells (Nunc 384 shallow well plate, black, 264705) were pre-blocked by incubation with CMG-binding buffer supplemented with 1 mg/ml BSA to avoid non-specific sticking of CMG and DNA templates to the wells during the assay. Blocking buffer was removed from a well, and 5 μL of CMG/DNA mixture was transferred into the well. 15 μL of ATP mix supplemented with 1.5 μM Oligo-5 and Oligo-10 was added to initiate unwinding. Starting immediately, fluorescence intensity was recorded on a PHERAstar FS (BMG Labtech) with excitation and emission wavelengths of 640 and 680 nm, respectively. Data was acquired at 25°C with 5 s intervals for 50 min with 10 flashes/measurement. Measured signal values were normalized and plotted against time.

#### Fitting and Normalization

To normalize measured signal values obtained from fluorescence-based unwinding assays, individual datasets were fit to the function described in Donmez and Patel, (2008) and Lucius et al., (2003). Briefly, we used Equation 1 given below for integer values of m:

(1) $$f_{ss}\left(t\right)=1-\sum_{r=1}^{m}\frac{k_{obs}t^{\left(r-1\right)}}{\left(r-1\right)!}e^{-k_{obs}t}$$

where $f_{ss}\left(t\right)$ is time course of unwinding, m is the number of steps, $k_{obs}$ is the observed unwinding rate, and t is time. Time-dependent fluorescence intensities from unwinding assays were fit for m = 2 due to the presence of a lag phase at early time points, which leads to the Equation 2:

(2) $$f_{ss}\left(t\right)=1-\left(1+k_{obs}t\right)e^{-k_{obs}t}$$

#### Single-Molecule Unwinding Assays

Single-molecule DNA unwinding assays were performed using PEG-biotin-functionalized microfluidic flow chambers prepared as described in Yardimci et al., (2012b). To cover surface with SA, 100 μL of 0.2 mg/ml SA in PBS was drawn into the microfluidic flow chamber using a syringe pump (Harvard Apparatus) and incubated for 20 min. The flow chamber was extensively washed with blocking buffer (20 mM Tris-HCl pH 7.5, 50 mM NaCl, 2 mM EDTA, 0.2 mg/ml BSA) to remove excess SA. For immobilization of DNA, ∼40 pM of substrate was subsequently injected in blocking buffer and incubated for 10 min. The channel was washed with blocking buffer to remove unbound DNA molecules and equilibrated with 100 μL of CMG binding buffer supplemented with 0.1 mM ATPγS. 40 nM of *Dm*CMG in 20 μL of CMG binding buffer supplemented with 0.1 mM ATPγS was injected and incubated for 20 min. To initiate DNA unwinding, 50 μL of ATP mix supplemented with 15 nM EGFP-RPA (∼0.07 mg/ml) was introduced and incubated for ∼40 min while collecting images with 10 s intervals.

Imaging was performed on an objective-type TIRF configuration using an inverted microscope (Ti-E, Nikon) equipped with a 100X oil objective (HP Apo TIRF, N.A. = 1.49, Nikon). Fluorescence intensity of EGFP-RPA was recorded with excitation wavelength of 488 nm at 100 ms exposure. Images were acquired using an Andor iXon 897 back- illuminated electron-multiplying CCD camera (Andor Technology).

#### Replication Assays with *Xenopus* Egg Extracts

*Xenopus* egg extracts were prepared as described in Lebofsky et al., (2009). Plasmid bearing either clk-SA or MH was first incubated with 6 μM LacI at 37.5 ng DNA/μl for 40 min at room temperature. Next, HSS (final concentration of 7.5 ng DNA/μl HSS) was added, and the reaction was further incubated for 30 min for origin licensing. Subsequently, two volumes of NPE was added together with [α-^32^P]dATP to initiate replication and label nascent DNA strands. At indicated time points, reactions were stopped by addition of 10 μL of replication stop solution A (80 mM Tris-HCl pH 8.0, 5% SDS, 0.13% phosphoric acid, 10% Ficoll) supplemented with 1 μL of Proteinase K (20 mg/ml) (Roche) and incubated for 1 hour at 37°C. Replication intermediates were separated on 0.9% native agarose gel and visualized using phosphorimager. To analyze nascent leading strand products, 3-4 μL of each replication reaction was mixed with 10 volumes of Buffer R (50 mM Tris-HCl pH 7.5, 0.5% SDS, 25 mM EDTA). Replication intermediates were purified as previously described by Räschle et al., (2008). Purified DNA was digested with the indicated restriction enzymes, separated on 7% denaturing polyacrylamide gel, transferred to filter paper, dried, and visualized using a phosphorimager. The image presented in Figure 7 was transformed using the Log transform function in ImageJ (NIH, USA) to allow a better visualization of the nascent leading-strand products.

### Quantification and Statistical Data Analysis

Throughout the manuscript, the data are represented as average ± SD of pooled experiments unless otherwise stated. Prism (GraphPad Software, La Jolla, CA, USA) was used to plot all graphs presented and for statistical analysis in this study. P values were computed by one-way ANOVA and Tukey’s multiple comparison tests. P values less than 0.05 (^∗^) were considered significant. ImageJ was used to quantify band intensities in gel images. 16-bit images with ‘.gel’ extension were first linearized using LinearizeData command in ImageJ.

## Supplemental Information

Document S1. Figures S1–S7 and Tables S1 and S2

Document S2. Article plus Supplemental Information
