# Supplementary material for: Dynamics of the Eukaryotic Replicative Helicase at Lagging-Strand Protein Barriers Support the Steric Exclusion Model
Source: Cell Rep. 2019 Feb 19;26(8):2113–2125.e6. doi: 10.1016/j.celrep.2019.01.086 (PMC6381796; doi:10.1016/j.celrep.2019.01.086)
Supplement: Document S1. Figures S1–S7 and Tables S1 and S2 [file mmc1.pdf]

Cell Reports, Volume *26*

Supplemental Information

Dynamics of the Eukaryotic Replicative Helicase at Lagging-Strand Protein Barriers Support the Steric Exclusion Model

Hazal B. Kose, Nicolai B. Larsen, Julien P. Duxin, and Hasan Yardimci

Figure S1

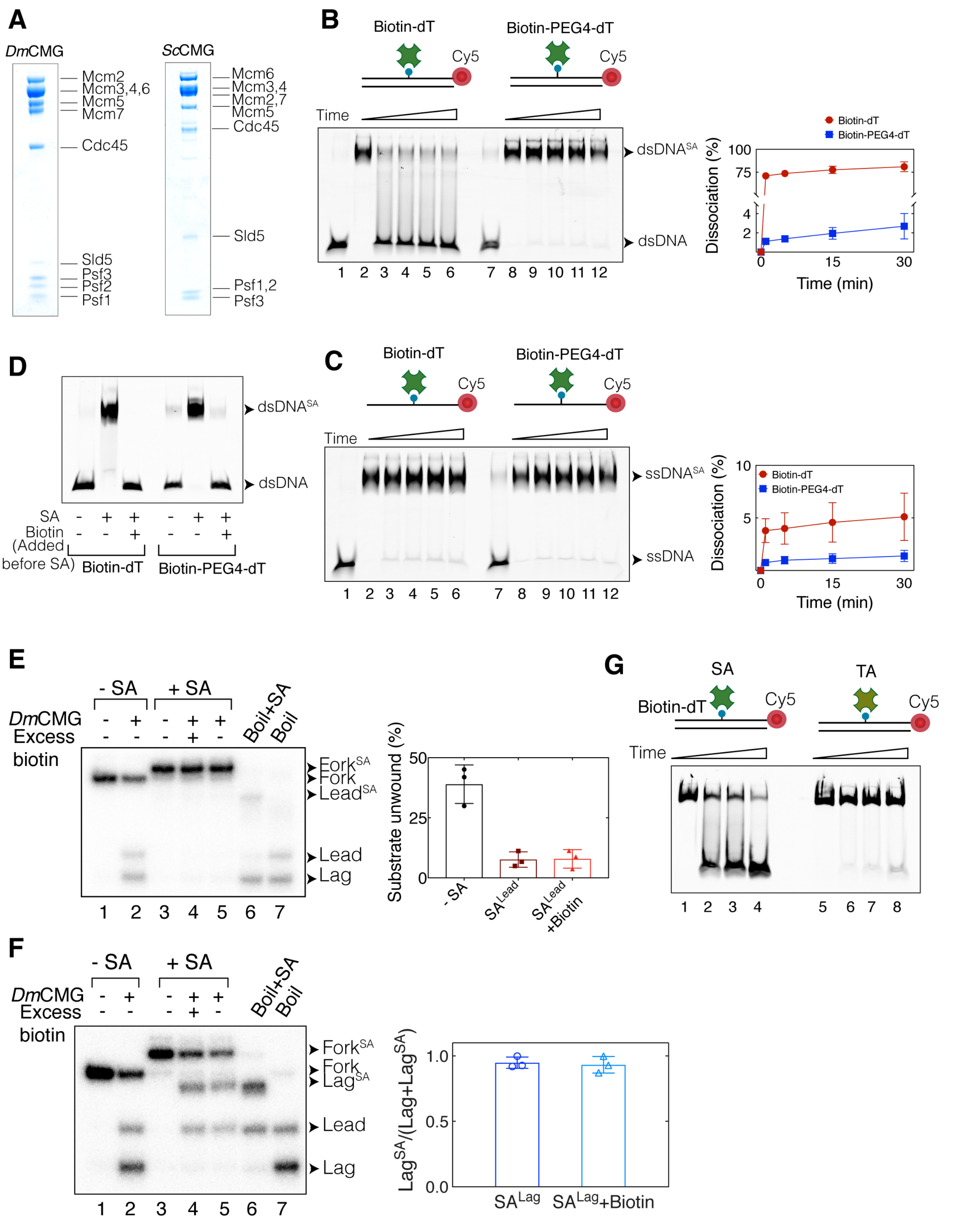

Figure S1. Recombinant CMG complexes and stability of biotin-streptavidin complex on DNA, Related to Figure 1.

(A) Purified recombinant *Dm*CMG and *Sc*CMG complexes were separated on 4-12% SDS polyacrylamide gels and stained with coomassie blue.

(B and C) Gel images showing spontaneous dissociation of SA from (B) dsDNA or (C) ssDNA containing either an internal biotin-dT (lanes 1-6) or biotin-PEG4-dT (lanes 7-12). DNA was labelled with Cy5 at the 3′ end of the biotin-modified strand. Right panels show the extent of SA dissociation at indicated time points upon incubation at 30°C with excess biotin. Samples in lane 1 and 7 do not include SA, in lanes 2 and 8 contain SA but not biotin. Lanes 3-6 and 9-12 correspond to 1, 5, 15, and 30 min of biotin incubation after SA binding. Each reaction included final concentrations of 0.5 mg/ml SA and 80 μM biotin. Data represented here are mean±SD from three independent experiments. Spontaneous SA dissociation in the presence of free biotin is greatly enhanced when the biotin-modified strand is annealed to its complementary strand suggesting that base pairing weakens the bio–SA interaction.

(D) SA failed to bind biotinylated duplex DNA templates when biotin was added before SA.

(E) *Dm*CMG-mediated unwinding of fork DNA modified with SA^{Lead} in the presence (lane 4) or absence (lane 5) of free biotin. Percentage of substrate unwound under different conditions is shown on the right panel. While SA^{Lead} almost completely inhibited unwinding, addition of excess free biotin did not increase the unwinding efficiency.

(F) *Dm*CMG-dependent unwinding of fork DNA modified with SA^{Lag} in the presence (lane 4) or absence (lane 5) of free biotin. The right panel shows the relative amount of SA-bound lagging-strand template (Lag^{SA}) with respect to total amount of lagging-strand template ($Lag+Lag^{SA}$) in the absence (lane 5) or presence (lane 4) of excess biotin. Each reaction contained 75 nM *Dm*CMG. Data represented here are mean±SD from three independent experiments.

(G) Spontaneous dissociation of SA (SA) or traptavidin (TA) from dsDNA containing an internal biotin-dT. Reactions in lanes 1 and 5 did not include excess biotin, while the rest contained 80 μM biotin. After addition of biotin, DNA was incubated at 30°C for 5, 15, or 30 min.

Figure S2

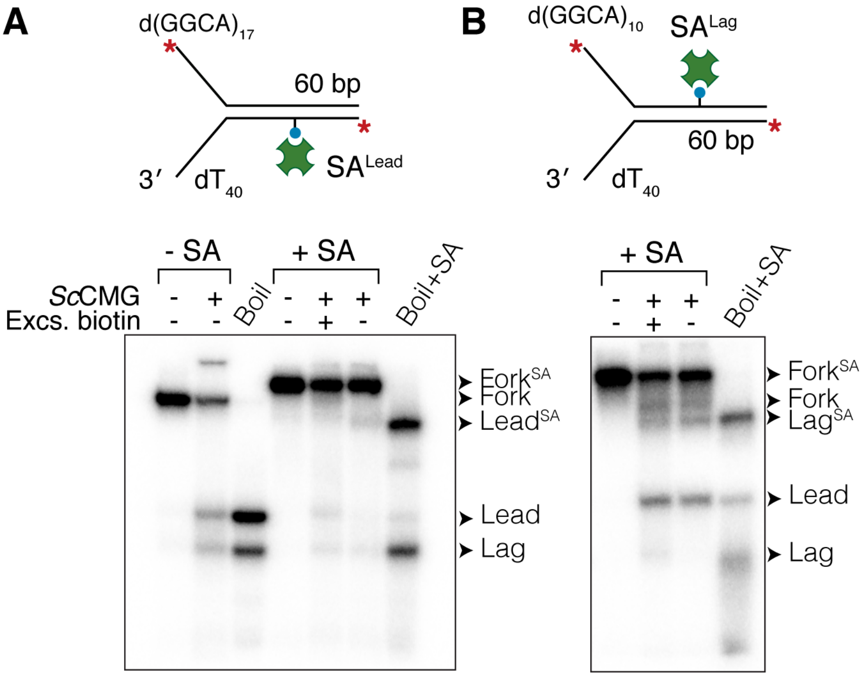

Figure S2. *Sc*CMG Does Not Displace Streptavidin from the Lagging-Strand Template, Related to Figure 2.

(A and B) *Sc*CMG-catalyzed unwinding of fork DNA templates containing (A) SA^{Lead} or (B) SA^{Lag} in the absence or presence of excess biotin. Intensity of the band corresponding to SA-bound lagging strand (Lag^{SA}) did not significantly change upon addition of excess biotin. The slight increase in streptavidin-free lagging strand-template (Lag) is most likely due to spontaneous dissociation of SA from fork DNA when challenged with free biotin. Fork substrates were labelled at both 5′-ends with ^{32}P. The radiolabel is shown as a red asterisk.

Figure S3

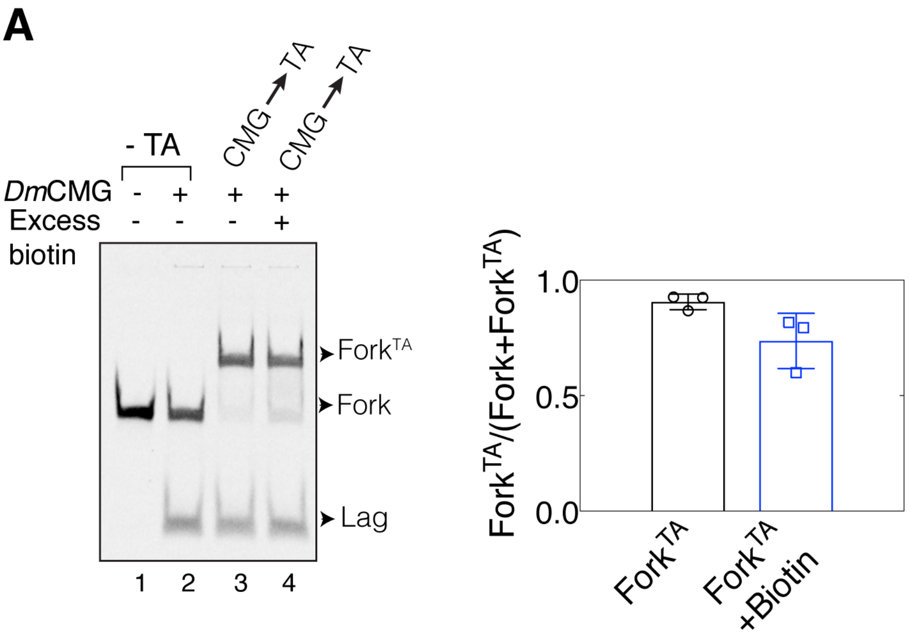

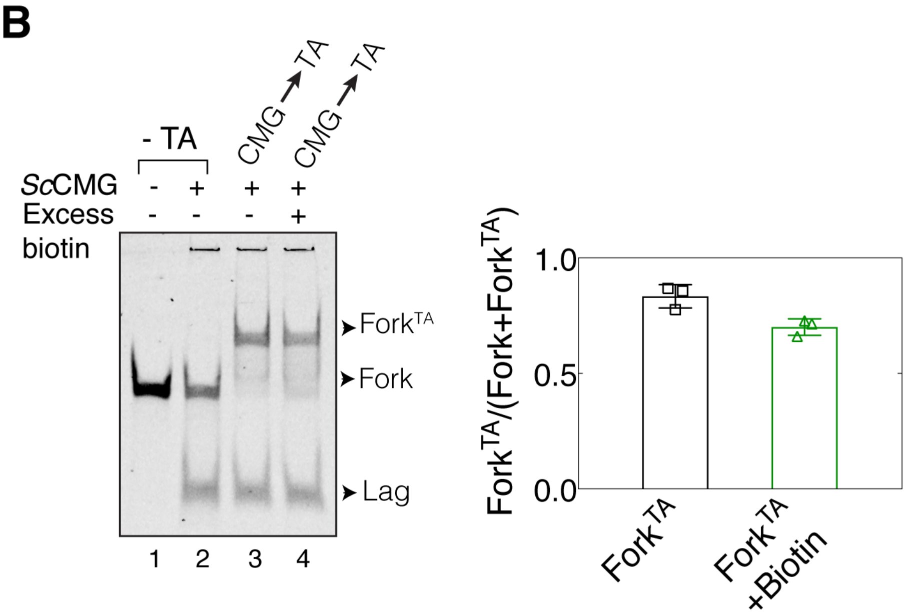

Figure S3. Traptavidin Efficiently Binds to CMG-Bound Fork DNA, Related to Figure 3.

(A and B) Unwinding of fork DNA bearing biotin-dT-TA^{Lag} by (A) *Dm*CMG or (B) *Sc*CMG in the absence (lane 3) or presence (lane 4) of excess biotin. Right panels show the fraction of fork DNA bound to TA (as measured by Fork^{TA}/(Fork^{TA}+Fork)) in the absence or presence of excess biotin. Addition of free biotin leads to only a minor decrease in the amount of TA-bound DNA most likely due the dissociation of TA. Data represented here are mean±SD from three independent experiments.

Figure S4

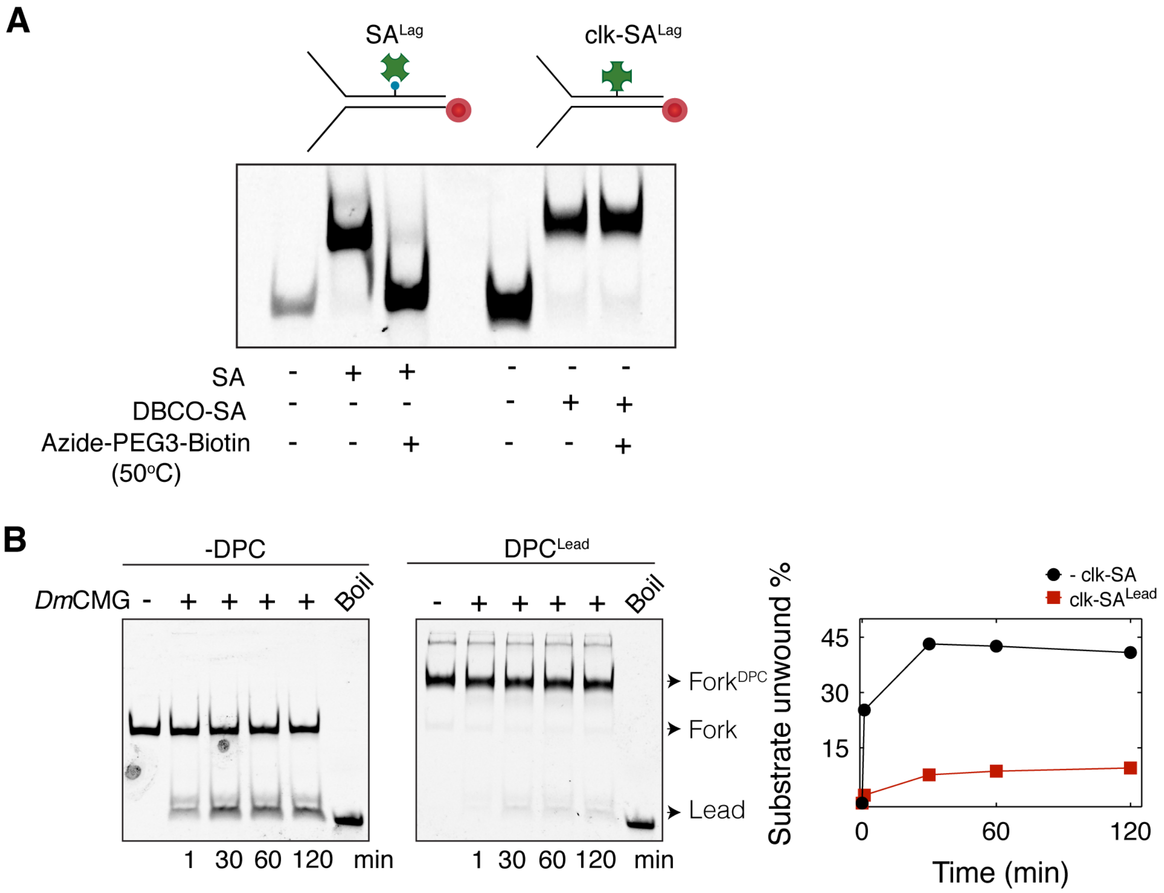

Figure S4. *Dm*CMG Cannot Efficiently Bypass DPC^{Lead} After Extended Periods of Time, Related to Figure 4.

(A) Stability of SA bound to Cy5-labeled fork DNA via biotin or covalently through copper-free click chemistry is measured by incubation at 50°C for 10 min in the presence of a bifunctional reagent, azide-PEG3-biotin, that competes with biotin and azide on DNA substrates.

(B) Time-course unwinding assays on unmodified and clk-SA^{Lead}-modified fork templates in the presence of 50 nM *Dm*CMG. Right panel shows percentage of unwound substrate in the absence or presence of clk-SA^{Lead} plotted against time.

Figure S5

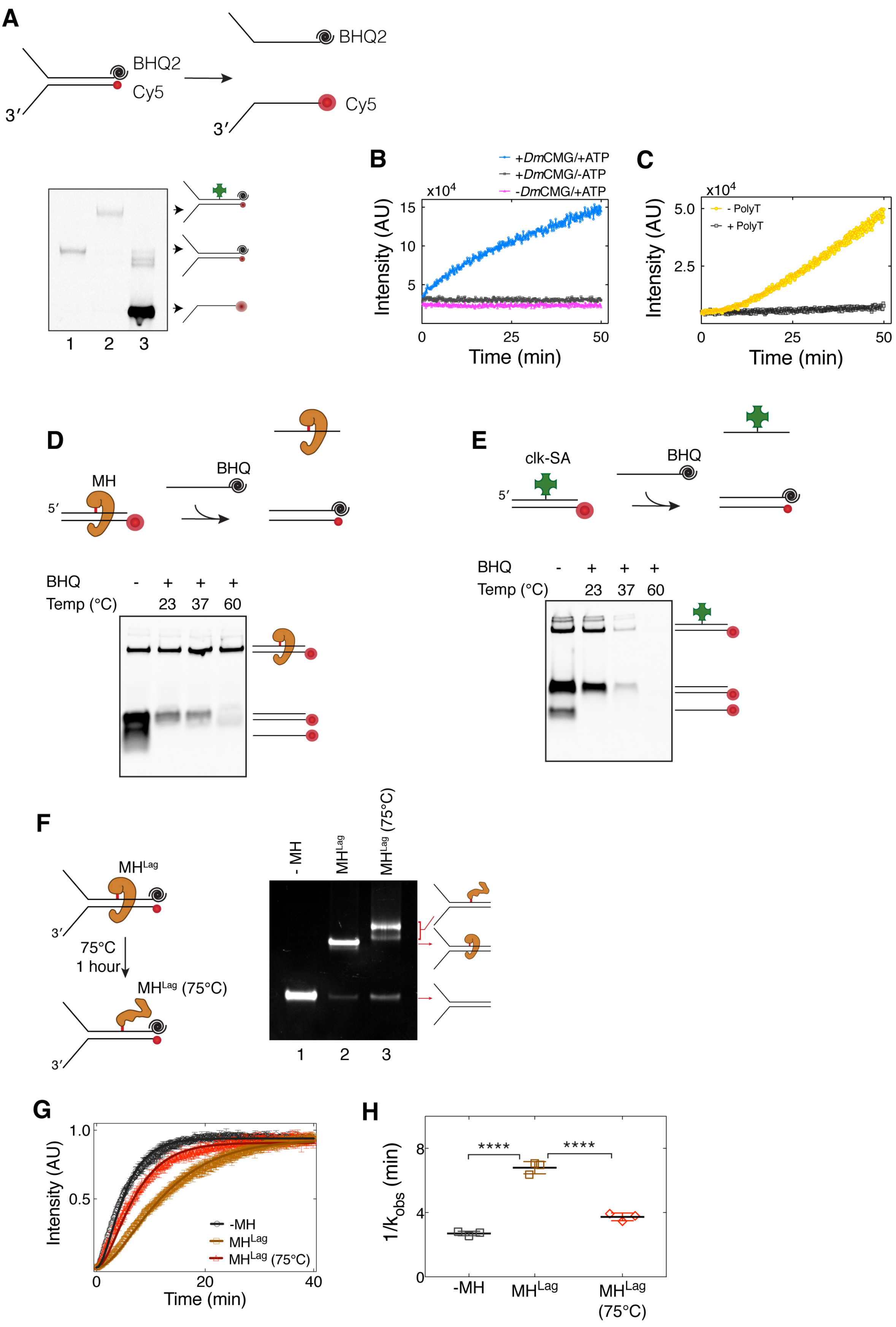
A
BHQ2
Cy5
3′
1 2 3
B
+DmCMG/+ATP
+DmCMG/-ATP
-DmCMG/+ATP
x10^4
Intensity (AU)
Time (min)
C
- PolyT
+ PolyT
D
MH
BHQ
5′
BHQ - + + +
Temp (°C) 23 37 60
E
clk-SA
F
MH^Lag
75°C
1 hour
MH^Lag (75°C)
- MH
1 2 3
G
-MH
H
1/k_obs (min)
****

Figure S5. Stalling of *Dm*CMG is Alleviated Upon Unfolding of MH^{Lag}, Related to Figure 5.

(A) Schematic representation of Cy5 fluorescence enhancement by conversion of dsDNA to ssDNA. (Lower panel) Naked (lane 1) and clk-SA^{Lag}-modified (lane 2) fork DNA containing Cy5 and BHQ2 on opposite strands were separated on native polyacrylamide gel. Upon heat denaturation of dsDNA, Cy5-modified strand exhibits higher fluorescence (lane 3).

(B) *Dm*CMG was pre-incubated with labelled fork DNA in the presence of ATPγS for 120 min. Unwinding was initiated by addition of ATP. Fluorescence time course exhibits intensity increase in the presence of *Dm*CMG and ATP (blue) indicating helicase-dependent fork unwinding. Omission of *Dm*CMG (pink) or ATP (black) results in no change in fluorescence as expected.

(C) When *Dm*CMG is mixed with fork DNA and ATP without pre-loading onto fork, a lag period in fluorescence signal increase, during which CMG binds to DNA, is observed (yellow). Presence of 40-nt poly-T oligonucleotide (1.5 μM final) in this assay prevents CMG to bind fork DNA substrate and subsequent unwinding (black).

(D and E) Thermal stability of duplex DNA substrates bearing (D) MH or (E) clk-SA. Substrates were incubated in the absence (lane 1) or presence (2, 3, and 4) of BHQ-labelled competitor oligo at indicated temperatures for 20 min. Melting of duplex DNA leads to liberation of the Cy5-labelled strand, its hybridization to the BHQ-labelled strand upon cooling, and disappearance of the fluorescent band.

(F) A schematic representation of MH^{Lag} unfolding upon 75°C treatment. On the right, naked (lane 1), MH^{Lag}-modified (lane 2), and heat-treated MH^{Lag}-modified (lane 3) fork DNA substrates were separated on native 8% polyacrylamide gel. Upon heat treatment, most DNA contained unfolded MHpaII (lane 3).

(G) Single turn-over fluorescence-unwinding assays performed with denatured-MH^{Lag} fork substrate (red) showed significantly less delay compared to substrates with native MH^{Lag} (brown). Unwinding kinetics of substrate lacking MH is shown in black. Solid lines represent fits to the Equation 2 (STAR Methods).

(H) Observed unwinding rate constants of substrates from (G). Data represented here are mean±SD from three independent experiments. $^{*}p < 0.05$, $^{**}p < 0.01$, $^{***}p < 0.001$.

Figure S6

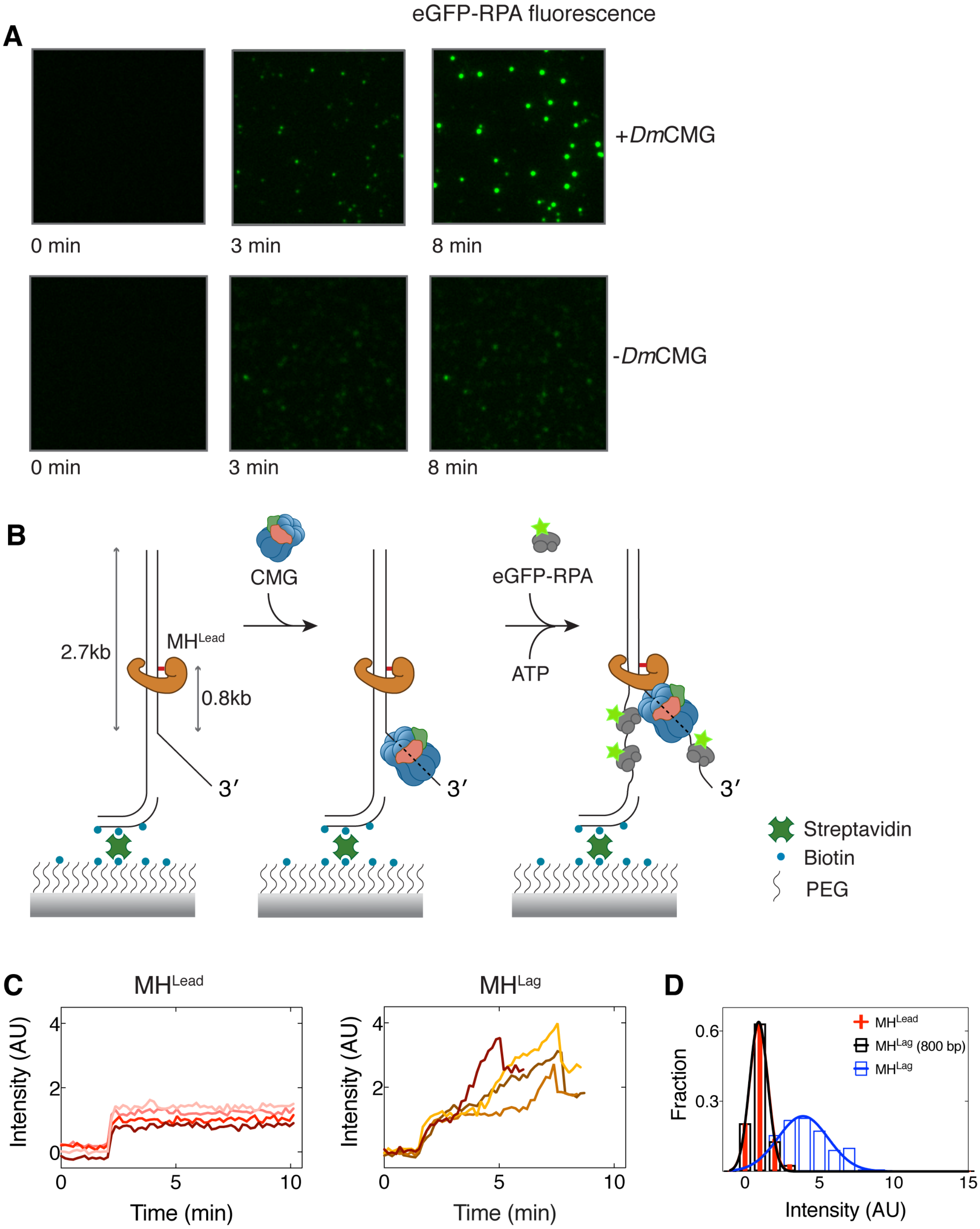

Figure S6. Fluorescence Signal Accumulation is Unwinding-Dependent, Related to Figure 6.

(A) Fluorescence images of a sample field of view at different time points showing accumulation of eGFP-RPA signal in the presence (top row) and absence (bottom row) of pre-incubation with *Dm*CMG. Time points are from the addition of eGFP-RPA into the flow chamber.

(B) Schematic representation of experimental approach with DNA substrate bearing MH on the leading-strand template (MH^{Lead}).

(C) Example unwinding traces of substrates with MH^{Lead} (left) or MH^{Lag} (right). Traces reach plateau upon reaching MH^{Lead} as CMG stops unwinding (depicted in B).

(D) Distribution of intensity levels measured at the peak point on fully unwound substrates with MH^{Lead} (red) and MH^{Lag} (blue), or at the transient stalling region in MH^{Lag} substrates (black). Average fluorescence accumulation on fully unwound MH^{Lag}-modified DNA is approximately ~3.3-fold higher than that on substrates with MH^{Lead}. Number of molecules are n(MH^{Lag})=109, n(MH^{Lead})=39, n(MH^{Lag} until pause)=13.

Figure S7

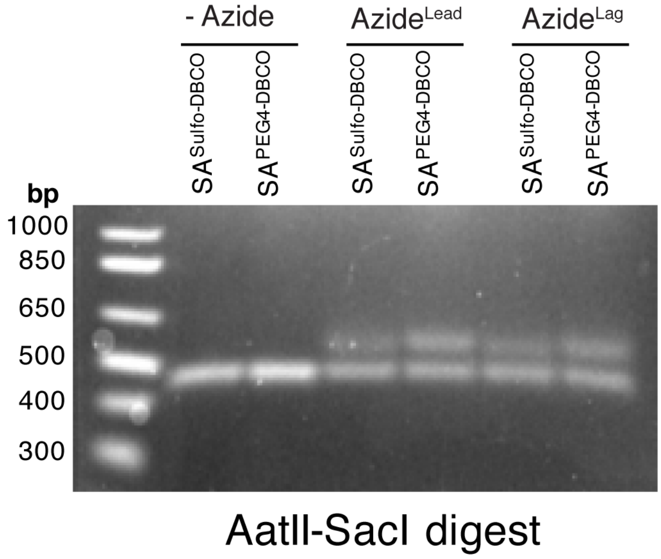

Figure S7. Covalent Conjugation of SA to Plasmid DNA, Related to Figure 7.

Azide-modified plasmid was crosslinked to SA that was functionalized either with DBCO-sulfo-NHS or DBCO-PEG4-NHS. To measure the crosslinking efficiency, plasmid was digested with AatII and SacI freeing a 500 bp fragment containing the modified base. SA functionalized with DBCO-PEG4 led to approximately 50% crosslinking efficiency of plasmids containing azide on either the leading- or the lagging-strand templates. Because SA modified with DBCO-sulfo was less proficient in click conjugation, we used plasmids containing SA with DBCO-PEG4 in replication assays.

Table S1. Sequences of oligonucleotides used in this study. Related to STAR Methods.

Oligo Name	Sequence
Oligo-amn-1	GGCTCGTTTTACAACGTCGTGCTGAGGTGA[Amine-dT]ATCTGCTGAGGCAATGGGAATTCGCCAACCTTTTTTTTTTTTTTTTTTTTTTTTTTTTTTTTTTTTTTT
Oligo-amn-2	GGCAGGCAGGCAGGCAGGCAGGCAGGCAGGCAGGCAGGCAGGTTGGCGAATTCCCATTGCCTCAGCAGA[Amine-dT]ATCACCTCAGCACGACGTTGTAAAACGAG
Oligo-amn-3	TCAGCA[Amine-dT]CCGGTACC
Oligo-azide-1	[5'-Phos]TCAGCA[Azide-dT]CCGGTACC
Oligo-azide-2	[5'-Phos]TGAGG[Azide-dT]ACCGGATGC
Oligo-azide-3	[5'-Phos]TCAGCAGGTCCGGCTTAAGCCTTA[Azide-dT]AAAGGTACC
Oligo-azide-4	[5'-Phos]TGAGGTACCTTTA[Azide-dT]AAGGCTTAAGCCGGACCTGC
Oligo-bio-1	GGCAGGCAGGCAGGCAGGCAGGCAGGCAGGCAGGCAGGCAGACGCTGCCGAA[Biotin-dT]TCTGGCTTGCTAGGACATTACAGGATCGTTCGGTCTC
Oligo-bio-Cy5	GGCGAATTCCCATTGCCTCAGCA[Biotin-dT]CCGGTACCTCAGCACGACGTTGTAAA[3'-Cy5]
Oligo-BHQ2-1	[5'-Phos]TCAGCACGACGTTGTAAAACGAGC[3'-BHQ2]
Oligo-BHQ2-2	ACAGCATCCGGTACCC[3'-BHQ2]
Oligo-Cy5-1	[5'Cy5]GCTCGTTTTACAACGTCGTGCTGAGGTACCGGATGCTGAGGCAATGGGAATTCGCCAACCTTTTTTTTTTTTTTTTTTTTTTTTTTTTTTTTTTTTTTTT
Oligo-Cy5-2	[5'Phos]CCATTGCCTCAGCATCCGGTACCTCAGCACGACGTTGTAAAACGAGC[3'-Cy5]
Oligo-Cy5-3	[5'-Phos]TCAGCACGACGTTGTAAAACGAGCCA[3'-Cy5]
Oligo-Cy5-4	[5'Cy5]GAGACCGAACGATCCTGTAATGTCCTAGCAAGCCAGAATTCGGCAGCGTCTTTTTTTTTTTTTTTTTTTTTTTTTTTTTTTTTTTTTTTT
Oligo-Cy5-5	[5'-Cy5]GGGTACCGGATGCTGT
Oligo-fluo-1	[5'-Phos]TCAGCATC[5-Fluoro-2'-dC]GGTACC
Oligo-fluo-2	[5'-Phos]TCAGCATCCGGTAGCTACTCAATC[5-Fluoro-2'-dC]GGTACC
Oligo-fluo-3	[5'-Phos]TGAGGTAC[5-Fluoro-2'-dC]GGATTGAGTAGCTACCGGATGC
Oligo-1	GGCAGGCAGGCAGGCAGGCAGGCAGGCAGGCAGGCAGGCAGGCAGGCAGGCAGGCAGGCAGGCAGGCAGGTTGGCGAATTCCCATTGCCTCAGCAGATATCACCTCAGCACGACGTTGTAAAACGAG
Oligo-2	GGCTCGTTTTACAACGTCGTGCTGAGGTGATATCTGCTGAGGCAATGGGAATTCGCCAACCTTTTTTTTTTTTTTTTTTTTTTTTTTTTTTTTTTTTTTTT
Oligo-3	[5'Phos]TGAGGCAATGGGAATTCGCCAACCTTTTTTTTTTTTTTTTTTTTTTTTTTTTTTTTTTTTTTTT
Oligo-4	GGCAGGCAGGCAGGCAGGCAGGCAGGCAGGCAGGCAGGCAGGTTGGCGAATTCCCATTGCC
Oligo-5	TTTTTTTTTTTTTTTTTTTTTTTTTTTTTTTTTTTTTTTT
Oligo-6	[5'-Phos]TCAGCACGACGTTGTAAAACGAGC
Oligo-7	GCTCGTTTTACAACGTCGTGC
Oligo-8	GGCAGGCAGGCAGGCAGGCAGGCAGGCAGGCAGGCAGGCAGGTTGGCGAATTC
Oligo-9	GGCTCGTTTTACAACGTCGTGCTGAGGTACCGGATGCTGAGGCAATGGGAATTCGCCAACCTTTTTTTTTTTTTTTTTTTTTTTTTTTTTTTTTTTTTTTT
oligo-10	GGATGCTGAGGCAATGGGAATTCGCCAACC

oligo-11	TTTACAACGTCGTGCTGAGGTACCGGATGCTGAGGCAATGGGAATTCGCC
Oligo-12	ATGCCGGGAGCAGACAAGCCCGTC
Oligo-13	TTTCTTGTATAGCAGTGCAGCTTT
Oligo-14	GGCTTACATTTTTTT
Oligo-15	GGCAAGAGCAACTCGGTCGCCGCATACACTATTCTCAGAATGACTTGGTTAT GTAAGCC
Oligo-16	AACCAAGTCATTCTGAGAATAGTGTATGCGGCGACCGAGTTGCTCTTGCCGG CAGGCAGGCAGGCAGGCAGGCAGGCAGGCAGGCAGGCATGCTCTTTACAA CCGGTAGACTGCTTCAGGGAACGATGTGCTGTGTACAGAGCTCC
Oligo-17	GTACACAGCACATCGTTCCCTGAAGCAGTCTACCGGTTGTAAAGAGCATTTT TTTTTTTTTTTTTTTTTTTTTTTTTTTTTTTTTTTTTTTTTTTTTTTTTTTTTTTTTT TTTTTTTT
Oligo-18	AGAGCTCCTCAGCGAGAGCGCACGAGGGAGC
Oligo-19	TAGCGCCTCAGCCTGTTCCGACCCTGCCGCTTA
Oligo-20	TAGCGCCTCAGCTCTGACAACGATCGGAGGACC
Oligo-21	AGAGCTCCTCAGCAGTAAGTTGGCCGCAGTGTTATCAC
Oligo-22	TTTTTTTGGAGCTCT
Oligo-23	TTTTTTGGCGCTA

Table S2. Composition of DNA substrates and figures associated with each substrate. Related to STAR Methods.

Substrate Name	Oligonucleotides	Associated Figures
Fork-biotin-PEG4^{Lead} (Radiolabelled)	Oligo-1 and Oligo-amn-1	Figures 1, 2, S1, and S2
Fork-biotin-PEG4^{Lag} (Radiolabelled)	Oligo-2 and Oligo-amn-2	Figures 1, 2, S1, and S2
Fork-biotin^{Lag} (Cy5 labelled)	Oligo-bio-1 and oligo-Cy5-4	Figures 3 and S3
Fork-biotin-PEG4^{Lag} (Cy5 labelled)	Oligo-4, oligo-9, oligo-Cy5-3 and oligo-amn-3	Figure S4
Fork-clk-SA^{Lead} (Cy5 labelled)	Fork end: Oligo-3 and Oligo-8. Duplex end: Oligo-Cy5- 2, Oligo-azide-2 and Oligo-7	Figures 4 and S4
Fork-clk-SA^{Lag} (Cy5 labelled)	Oligo-Cy5-1, Oligo-4, Oligo-azide-1 and Oligo-6	Figures 4 and S4
Fork-clk-SA^{Lag} (Cy5 and BHQ2 labelled)	Oligo-Cy5-1, Oligo-4, Oligo-azide-1 and Oligo- BHQ2	Figure 5
Fork-MH^{Lag} (Cy5 and BHQ2 labelled)	Oligo-4, Oligo-Fluo-1, Oligo-Cy5-1, Oligo-BHQ2-1	Figure 5
2.7 kb-MH^{Lag} (Biotinylated)	Oligo-12, Oligo-13, Oligo-14, Oligo-15, Oligo-16, Oligo-17, Oligo-18, Oligo-19, Oligo-22, Oligo-23, Oligo-Fluo-1 and pHY39	Figure 6
2.7 kb-MH^{Lead} (Biotinylated)	Oligo-12, Oligo-13, Oligo-14, Oligo-15, Oligo-16, Oligo-17, Oligo-20, Oligo-21, Oligo-22, Oligo-23, Oligo-Fluo-1 and pHY39	Figure S6
Duplex-biotin (Cy5 labelled)	Oligo-11 and oligo-bio-Cy5	Figure S1
Duplex-biotin-PEG4 (Cy5 labelled)	Oligo-11 and oligo-amn-Cy5	Figure S1
Duplex-5FdC (Cy5 labelled)	Oligo-Fluo-1, Oligo-Cy5-5	Figure S5
Duplex-Azide (Cy5 labelled)	Oligo-Azide-1, Oligo-Cy5-5	Figure S5
